# Differences and correlation analysis of feeding habits and intestinal microbiome in *Schizopygopsis microcephalus* and *Ptychobarbus kaznakovi* in the upper reaches of Yangtze River

**DOI:** 10.3389/fmicb.2025.1513401

**Published:** 2025-03-11

**Authors:** Xinyu Wang, Jiahui Hao, Cunfang Zhang, Ping Zhu, Qiang Gao, Dan Liu, Miaomiao Nie, Junmei Jia, Delin Qi

**Affiliations:** ^1^State Key Laboratory of Plateau Ecology and Agriculture, Qinghai University, Xining, China; ^2^College of Eco-Environmental Engineering, Qinghai University, Xining, China; ^3^Northwest Institute of Plateau Biology, Chinese Academy of Sciences, Xining, China

**Keywords:** *Schizopygopsis microcephalus*, *Ptychobarbus kaznakovi*, intestinal microbiota, DIETS, ecological niche, correlation analysis

## Abstract

**Background:**

The intestinal microbiota has co-evolved with the host to establish a stable and adaptive microbial community that is essential for maintaining host health and facilitating food digestion. Food selection is a critical factor influencing variations in gut microbial composition, shaping gut microbiome communities, and determining the ecological niches of fish.

**Methods:**

In this study, high-throughput amplicon sequencing of 16S rRNA and 18S rRNA was utilized to compare the dietary and gut microbial differences between *Schizopygopsis microcephalus* and *Ptychobarbus kaznakovi*, both collected from the same sites in the Tuotuo River and Tongtian River, which are tributaries of the Yangtze River. We compared the microbial community structure, diet composition, and diversity between the two fish species using various analytical methods, including LefSe, α-diversity and β-diversity analyses. Additionally, we constructed co-occurrence networks to determine their correlations.

**Results and discussion:**

The alpha diversity results indicated that *S. microcephalus* exhibited higher intestinal microbiota and feeding diversity compared to *P. kaznakovi*. Furthermore, the beta diversity results revealed significant differences in both intestinal microbiota and eukaryotic communities between the two species. The dominant bacterial phyla in both *S. microcephalus* and *P. kaznakovi* included Proteobacteria, Firmicutes, Actinobacteriota, Chloroflexi, and Verrucomicrobiota; however, Firmicutes was significantly more abundant in *P. kaznakovi* (*P* = 0.006), while Actinobacteriota was significantly higher (*P* = 0.019) in *S. microcephalus* at the phylum level. The primary food sources for *S. microcephalus* and *P. kaznakovi* were identified as Streptophyta (54.41%, 77.50%) and Cercozoa (8.67%, 1.94%), with Bacillariophyta (25.65%) was also the main food of constituting a major component of the diet for *S. microcephalus*. These differences suggested that *S. microcephalus* and *P. kaznakovi* occupy distinct dietary niches. To further explore the relationship between gut microbiota and feeding habits, we identified significant correlations between various food components and the gut microbial community through co-occurrence networks. This study enhances our understanding of the co-evolution and co-adaptation between host gut microbiota and feeding behaviors in sympatric fish species.

## 1 Introduction

Microorganisms generally coexist with plants, animals, and other organisms, with plant roots and animal intestines hosting the highest diversity of species (Wiriya et al., 2020). Gut microbes are often regarded as virtual organs within the host body. Although our understanding of how gut microbiota influences host fitness in animals remains limited, the significance of microbiota in host health has been well-established through various culturing methods. They play a crucial role in host diet, digestion, immunity, and environmental adaptation (Diwan et al., 2022). Research on fish and other animals has demonstrated that the diversity and abundance of commensal microbes are influenced by factors such as genetics, sex, host diet, developmental stage, aging, temperature, geographical distance, phages, and others (Jiang et al., 2020; Wei et al., 2020; Donati et al., 2022; Wei et al., 2022). Diet, in particular, is recognized as a decisive factor affecting the gut microbiome. Previous studies have revealed that the differences in gut microbes are particularly pronounced among carnivores, omnivores, and herbivores. The diversity of gut microbiota in wild animals is significantly higher than that in farmed animals (Uren Webster et al., 2018; Gibson et al., 2019), and there is a trend of increasing gut microbiome diversity from carnivores to omnivores to herbivores (Ley et al., 2008), leading to the formation of specific dominant bacteria in each animal group. In ants, they are categorized into herbivorous and carnivorous types. Herbivorous ants primarily feed on plant secretions and SAP (the fluid containing water, nutrients, and sugars in plants). The herbivorous ants of the genus host unique symbiotic bacteria, including *Burkholderia, Pseudomonas, Rhizobia, Verrucomicrobiales*, and *Xanthomonadales*, which possess the potential for symbiotic nitrogen fixation (Russell et al., 2009). Similar phenomena have also been observed in the study of cold-water fish, where significant differences and a downward trend in the relative abundances of Cyanobacteria and Plantaginaceae were noted across herbivorous, omnivorous, and carnivorous species. In contrast, the relative abundance of *Brevundimonas* exhibited an upward trend from herbivorous to omnivorous and then to carnivorous fish (Xu et al., 2022). Furthermore, both phylogeny and diet collectively influence the gut microbiota, and studying the structure of gut microbiota can yield insights into the evolutionary relationships among animals (Delsuc et al., 2014). There is evidence of evolutionary convergence in the gut microbes of animals with similar diets, suggesting that diet is a primary driver at larger scales (Muegge et al., 2011). For example, the bacterial composition found in the gut of giant pandas resembles that of other members of Ursidae, allowing researchers to investigate the evolutionary processes of both species through their intestinal flora (Groussin et al., 2017).

As aquatic vertebrates, fish exhibit a digestion process, nutrient uptake, and energy acquisition that can be significantly influenced by their gut microbial community (Parris et al., 2016; Bird et al., 2019; Xu et al., 2019; Liu et al., 2021). The study of gut microbes not only enhances our understanding of the changes in these communities during the evolution of organisms but also reveals the critical role of the microbiota in host nutritional physiology and foraging behavior (Trevelline and Kohl, 2022). However, compared to terrestrial animals, fish microbiotas are more susceptible to influences from host phylogeny, diet, environmental factors, and planktonic microbiota (Restivo et al., 2021). Recent studies have demonstrated that geographic distance also contributes to species divergence, which is accompanied by changes in gut microbiota (Liu et al., 2022). Consequently, it can be anticipated that the gut microbiome of fish undergoes alterations with environmental transitions (Restivo et al., 2021). Additionally, fish inhabit a unique environment, resulting in gut microbes that differ from those of terrestrial organisms. The intestinal microbiota can be classified as either indigenous (colonizing the epithelial surface or microvilli) or transient (present in the lumen and passing through the gut), with the latter largely dependent on diet (Ringø et al., 2015). Dietary preferences are influenced by various factors, including the developmental stage of individuals, seasonal changes, environmental shifts, and the host itself. Juvenile fish tend to prey on zooplankton, and as they grow, their dietary range expands (Sánchez-Hernández et al., 2022). Seasonal changes indirectly affect water temperature and light conditions, thereby influencing the abundance and types of available fish food. For instance, some fish increasing their consumption of zooplankton and aquatic insects (Santos et al., 2020), suggesting that environmental shifts compel fish to alter their food choices (Kelly et al., 2021).

Schizothorax represents a common biological group within the aquatic ecosystem of the Qinghai-Tibet Plateau. *Schizopygopsis microcephalus* (*S. microcephal*us) and *Ptychobarbus kaznakovi* (*P. kaznakovi*) occupy significant positions in the food chain of this ecosystem, playing a crucial role in maintaining ecological stability. Historically, these two species have served as important food sources for local residents; however, they currently face population threats due to their low numbers and slow growth. In recent years, the complete mitochondrial genomes of *S. microcephalus* (Li et al., 2016) and *P. kaznakovi* (Wu et al., 2017) have been sequenced. While most studies on fish in the Qinghai-Tibet Plateau have primarily focused on genomic adaptation and transcription in response to the complex environment (Wang et al., 2019), it is essential to recognize that the unique conditions of the Tibetan Plateau also contribute to the development of a complex gut microbiota and the low-oxygen environment plays a significant role in shaping the gut microbiota (Qi et al., 2012). Both *S. microcephal*us and *P. kaznakovi* belong to the subfamily Schizothoracinae. Specifically, *S. microcephalus* is classified under the genus *Schizopygopsis* and is primarily found in the high-altitude rivers and lakes of the Qinghai-Tibet Plateau, where it feeds on benthic diatoms, plant debris, and aquatic insects (Wu and Wu, 1992). This species is designated as a locally protected fish in Qinghai Province. Conversely, *P. kaznakovi* belongs to the genus *Ptychobarbus* and primarily inhabits the wide valley sections of the Jinsha River and the Yalong River in the upper reaches of the Yangtze River on the Qinghai-Tibet Plateau. Its diet mainly consists of aquatic insects and chironomid larvae, along with a variety of diatoms (Wu and Wu, 1992; Wang, 2015). This species is classified as a second-class protected animal in China. Therefore, studying their gut microbiota and feeding behavior is crucial for identifying their critical habitats and food resources, as well as understanding their adaptability to the plateau environment, which can positively impact their conservation.

Both *S. microcephal*us and *P. kaznakovi* inhabit the source of the Yangtze River, with the Tuotuo River and Tongtian River serving as their shared habitats. These two rivers exhibit unique environmental conditions, including a scarcity of food resources. Furthermore, the river basins are relatively unpolluted, providing excellent ecological conditions. To investigate the relationship between feeding habits and gut microbes, we analyzed the characteristics of intestinal microbes and feeding behaviors in *S. microcephalus* and *P. kaznakovi*, which coexist in sympatric distributions and occupy different ecological niches. This close relationship between gut microbes and feeding habits is also reflected in fish anatomy. Specifically, the intestinal length of herbivorous fish is more than three times that of carnivorous fish, which promotes the proliferation of anaerobic bacteria typical of herbivorous gut microbiota (Parata et al., 2020; Escalas et al., 2021). Therefore, studying of the interaction between the gut microbiome of fish and their feeding habits in the same waters of the Qinghai-Tibet Plateau offers insights for the development and utilization of the unique microbiota in the region, enhancing the nutrition and health of plateau fish, promoting large-scale breeding of plateau cold-water fish, and ultimately contributing to the conservation of plateau fish.

## 2 Materials and methods

### 2.1 Sample collection

*S. microcephalus* and *P. kaznakovi* were collected from two locations at the source of the Yangtze River: the branch of the Tuotuo River (E92°26′11″, N34°12′57″, H4518 m) in Tanggulashan Town, located in the southern region of Golmud City, and the Tongtian River (E92°26′3261″, N34°13′780″, H4413 m), Qinghai Province (Supplementary Figure S1). A total of five *S. microcephalus* and two *P. kaznakovi* samples were collected from the Tuotuo River, while three samples each of *S. microcephalus* and *P. kaznakovi* were collected from the Tongtian River. Both species were collected on sunny days in August 2021, using fish cages and trammel nets with a mesh size of 4 cm. Summer is the season when fish are more likely to access abundant food resources, thereby providing a better reflection of their survival status under food-rich conditions. We calculated the mean and standard deviation of weight and length for the captured individuals, collecting intestinal contents from 8 *S. microcephalus* with similar body weights (33 ± 10 g) and lengths (175.4 ± 69.6 mm) and 5 *P. kaznakovi* with similar body weights (54 ± 17 g) and lengths (188.8 ± 98.2 mm). The total length of each fish was measured from the tip of the snout to the end of the caudal fin using a ruler, while their weights were recorded using a digital scale. Each fish was euthanized with an overdose by 300 mg/L tricaine methane sulfonate (MS-222, Sigma) prior to sampling. The fish were then disintegrated, and the entire intestine was separated. It was subsequently washed three times with sterile PBS and all the intestinal contents were squeezed into a 2 mL cryotube. The sample was flash-frozen using liquid nitrogen and transported to the laboratory, where they were stored at −80°C. Finally, the samples were sent to Shanghai Meiji Biomedical Technology Co., Ltd. with dry ice for high-throughput sequencing analysis of the amplicon.

### 2.2 DNA extraction and PCR amplification

Genomic DNA was extracted from gut content samples using the E.Z.N.A.^®^ soil DNA Kit (Omega Bio-tek, Norcross, GA, U.S.) in accordance with the manufacturer's instructions. The quality of the DNA extract was assessed using a 1% agarose gel, while DNA concentration and purity were determined with a Nano Drop 2000 UV-vis spectrophotometer (Thermo Scientific, Wilmington, USA). For the PCR amplification of the 16S rRNA gene, the primers used were 338F (5′-ACTCCTACGGGAGGCAGCAG-3′) and 806R (5′-GGACTACHVGGGTWTCTAAT-3′), with an annealing temperature set at 55°C. For the 18S rRNA gene, the primers were 18SF (5′-TCYAAGGAAGGCAGCAGGCGC-3′) and 18SR (5′-GTTTCAGHCTTGCGACCATACTCC-3′), which had an annealing temperature of 61°C. Both PCR amplifications were performed with 35 cycles. The PCR mixtures consisted of 5 × TransStart FastPfu buffer (4 μL), 2 μL of 2.5 mM dNTPs, 0.8 μL of forward primer (5 μM), 0.8 μL of reverse primer (5 μM), 0.4 μL of TransStart FastPfu DNA Polymerase, 10 ng of template DNA, and ddH_2_O to a final volume of 20 μL. Each PCR reaction was performed in triplicate. The PCR products were extracted from a 2% agarose gel and purified using the AxyPrep DNA Gel Extraction Kit (Axygen Biosciences, Union City, CA, USA), following the manufacturer's guidelines and quantified with a Quantus™ Fluorometer (Promega, USA).

### 2.3 Illumina MiSeq sequencing and bioinformatics analysis

Purified amplicons were pooled in equimolar concentrations and paired-end sequenced on an Illumina MiSeq PE300 platform (Illumina, San Diego, USA) following the standard protocols established by Majorbio Bio-Pharm Technology Co. Ltd. (Shanghai, China). The raw sequencing reads for the 16S rRNA and 18S rRNA genes were demultiplexed, and quality control of the Fastq data was conducted using Trimmomatic and PEAR (Chen et al., 2018). The paired-end sequences were merged for merging based on their overlapping relationships using FLASH version 1.2.7 and PEAR version 0.96 (Magoc and Salzberg, 2011). The criteria for processing were as follows: (i) The 300 bp reads were truncated at any site where the average quality score fell below 20 over a 50 bp sliding window, with reads shorter than 50 bp discarded, and reads containing ambiguous characters also removed; (ii) Only overlapping sequences longer than 10 bp were assembled based on their overlap, with a maximum mismatch ratio of 0.2 in the overlap region. Reads that could not be assembled were discarded; (iii) Samples were distinguished according to barcodes and primers, with sequence direction adjusted for exact barcode matching, and allowing for a two-nucleotide mismatch in primer matching. Operational taxonomic units (OTUs) were clustered at a 97% similarity cut off using UPARSE version 7 (Stackebrandt and Goebel, 1994; Edgar, 2013). The uchime method in VSEARCH version 2.7.1 was employed to remove chimeric sequences from known databases, while the *de novo* method was used to eliminate sequences lacking reference. The taxonomy of each OTU representative sequence was analyzed using RDP Classifier version 2.2 (Wang et al., 2007) against the 16S rRNA and 18S rRNA databases, applying a confidence threshold of 0.7.

The pie charts and boxplots were visualized using online tools and Origin 2021 (https://www.originlab.com/). The α-diversity indices were calculated with the RStudio packages “reshape2”, “dplyr”, “stringr”, and “vegan”. To assess significant differences in composition and α-diversity indices between groups, the Wilcoxon rank-sum test was employed, with a threshold for q set at 0.05. Both the percentage accumulative histogram and PCoA analysis, based on Bray-Curtis distance and PERMANOVA tests, were generated using the Wekemo Bioincloud platform (https://www.bioincloud.tech/). LefSe and Venn diagrams were generated using the Tutools platform (https://www.cloudtutu.com/), with LefSe employing non-parametric factorial Kruskal-Wallis rank-sum tests and Wilcoxon rank-sum tests to identify biomarkers. Clustering barplots and the Mantel test were performed using Genescloud tools (https://www.genescloud.cn/), with clustering barplots based on the Bray-Curtis distance algorithm. The Mantel test utilized the Bray-Curtis distance algorithm, Spearman correlation algorithm, and Mantel test method. KEGG pathway maps were generated using the RStudio package “ggplot2”. Co-occurrence networks were constructed using the R packages “psych”, “reshape2”, “Hmisc”, and “ggplot2”, displaying the top 60 taxa based on abundance. Each dot in the networks represents a taxon, and the links indicate robust correlations, defined by Spearman's correlation coefficients (r) > |0.7| and a *P* value < 0.05. The co-occurrence networks were further visualized using Gephi-version 0.10.1 (https://gephi.org/).

## 3 Results

### 3.1 Overview of bacterial and diet composition of *S. microcephalus* and *P. kaznakovi*

To evaluate the usability of the sequencing data, we conducted an initial analysis. The rarefaction curves of gut bacterial communities for both fish species based on OTUs reached a saturation plateau, indicating that the sequencing depth was sufficient to represent the majority of microbial and foods species (Supplementary Figures S2A–D). Furthermore, the analysis of species accumulation demonstrated that the sample size was adequate for subsequent analysies (Supplementary Figures S2E, F).

**Figure 1:**
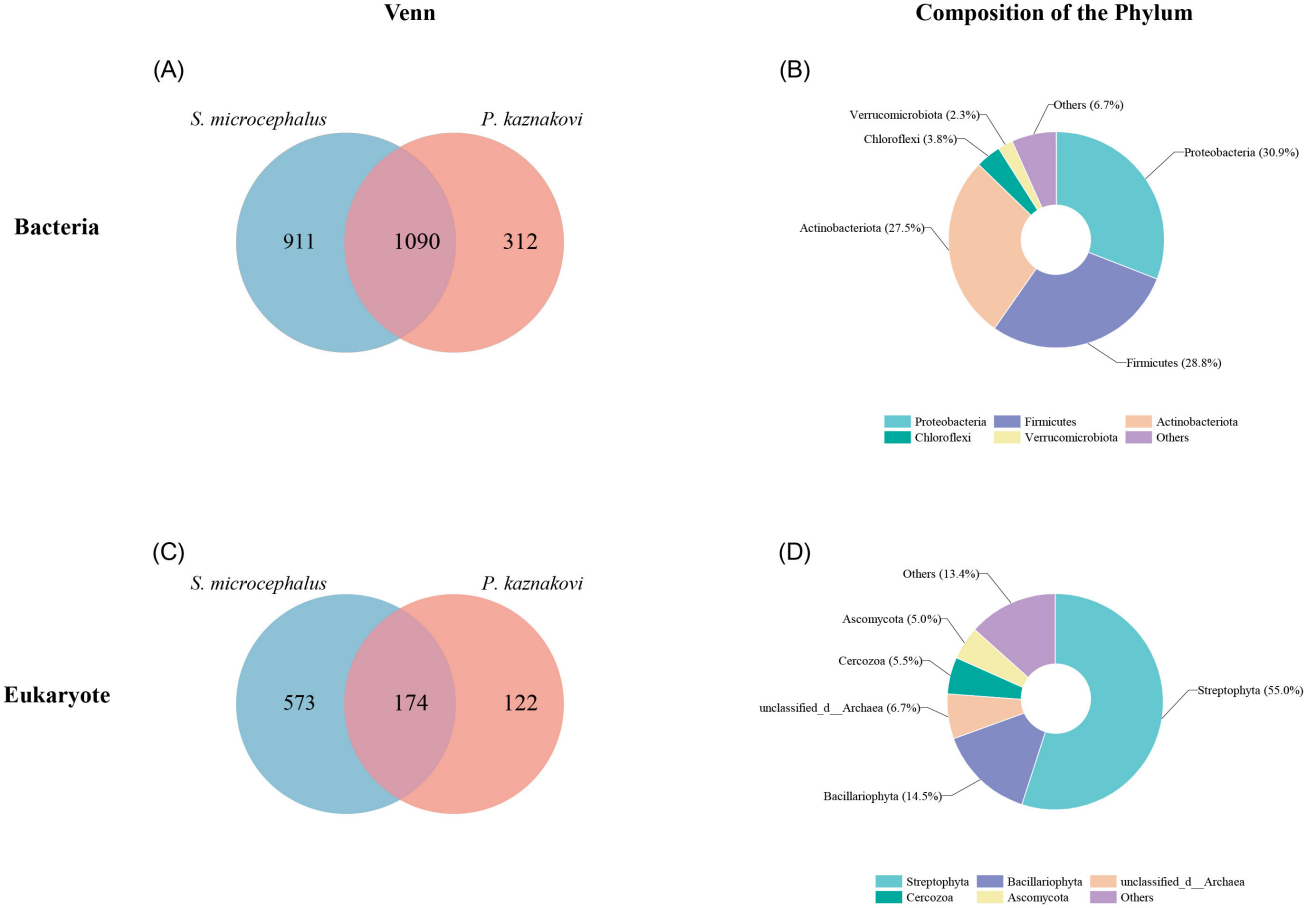
Overview of intestinal bacterial and diet composition of *S. microcephalus* and *P. kaznakovi*. **(A, B)** Venn diagram displaying the OTUs overlap of intestinal bacteria between *S. microcephalus* and *P. kaznakovi*, and the composition of the top five phyla. **(C, D)** Venn diagram displaying the OTUs overlap of diet between *S. microcephalus* and *P. kaznakovi*, and the composition of the top five phyla.

For 16S rRNA gene sequencing, an average of 56,471 reads was sequenced for each intestinal sample library. Following quality filtering and assembly, OTU clustering was conducted using a 97% similarity threshold. OTUs with a relative abundance sum of less than 0.0001 across all samples were excluded, resulting in a total of 2,313 OTUs. In the case of 18S rRNA gene sequencing, an average of 70,051 reads were sequenced for each intestinal sample library. After quality filtering and assembly, 869 OTUs were obtained at a 97% sequence similarity threshold. Due to the limited number of OTUs obtained, no further screening was performed. Overall, 2,313 OTUs were identified through our quality control process, with 1,090 OTUs shared between the two species, 911 OTUs were identified in *S. microcephalus* and 312 in *P. kaznakovi*, as determined by the 16S rRNA analysis (Figure 1A). The predominant bacterial phyla present in the combined samples of *S. microcephalus* and *P. kaznakovi* were Proteobacteria (30.9%), Firmicutes (28.8%), Actinobacteriota (27.5%), and Chloroflexi (3.8%) (Figure 1B). Similarly, in the 18S rRNA analysis, 869 OTUs were identified, with 174 OTUs shared between the two species, 573 OTUs unique to *S. microcephalus* and 122 unique to *P. kaznakovi* (Figure 1C). In the 16S rRNA analysis, the identified OTUs were classified into 33 phyla, 85 classes, 215 orders, 375 families, and 761 genera. In the 18S rRNA analysis, the identified OTUs were classified into 38 phyla, 93 classes, 170 orders, 237 families, and 301 genera. The predominant eukaryotic phyla included Streptophyta (55.0%), Bacillariophyta (14.5%), Cercozoa (5.5%), and Ascomycota (5.0%) (Figure 1D).

### 3.2 α-diversity and β-diversity analysis of intestinal microbiota and diet in *S. microcephalus* and *P. kaznakovi*

**Figure 2:**
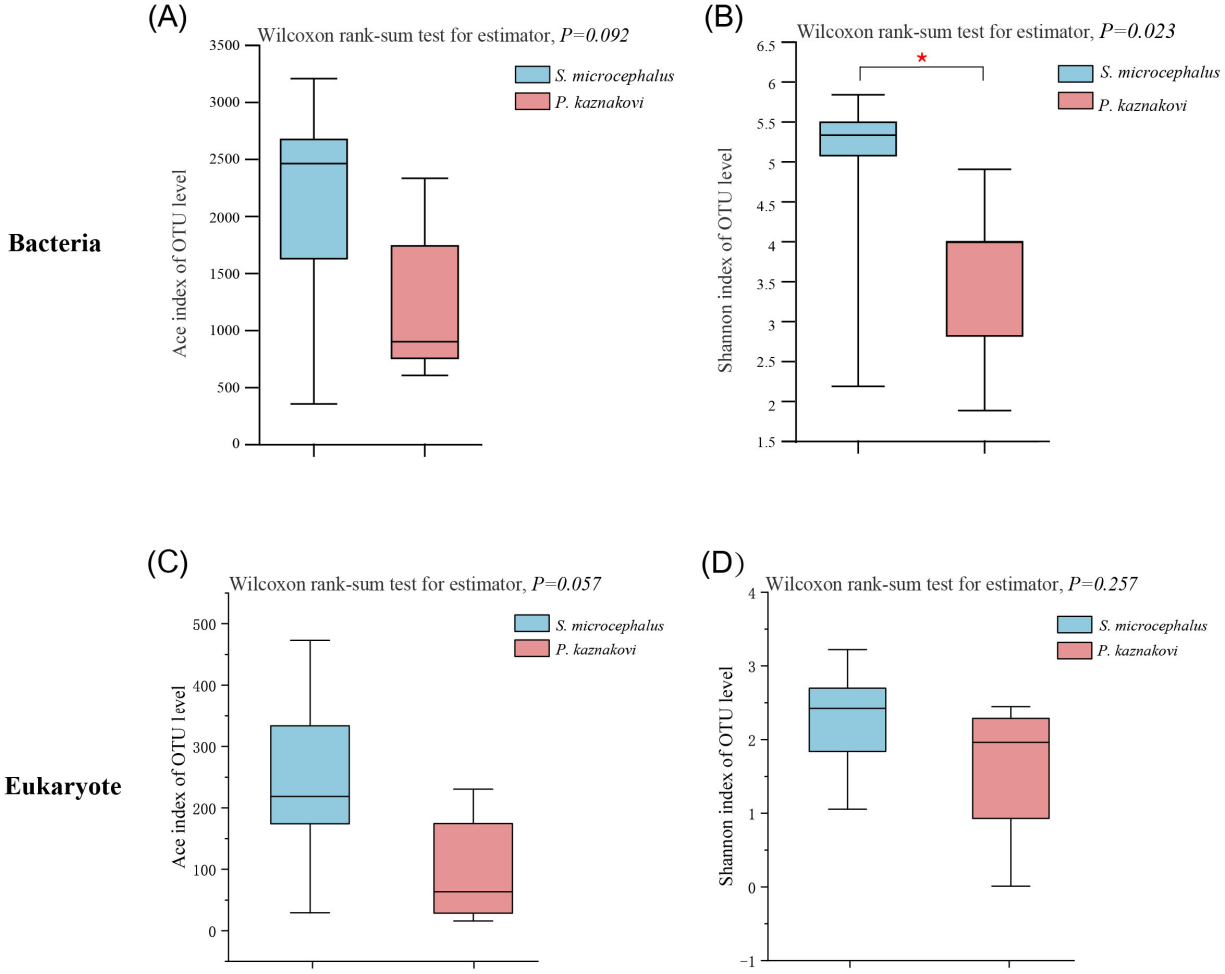
Comparison of alpha diversity between *S. microcephalus* and *P. kaznakovi*. The alpha diversity of bacteria **(A, B)** and eukaryote **(C, D)** were estimated by the Ace index and Shannon index. Asterisks indicate significant differences between *S. microcephalus* and *P. kaznakovi* according to a Wilcoxon rank-sum test: ^*^p < 0.05.

The alpha diversity analysis at the OTU level indicated that the Ace and Shannon indices for *S. microcephalus* were higher than those for *P. kaznakovi* in both bacterial and eukaryotic samples (Wilcoxon rank-sum test, *P* = 0.092, *P* = 0.023; *P* = 0.057, *P* = 0.257) (Figure 2). Notably, the Shannon index for bacteria in *S. microcephalus* was significantly higher than that for *P. kaznakovi*.

**Figure 3:**
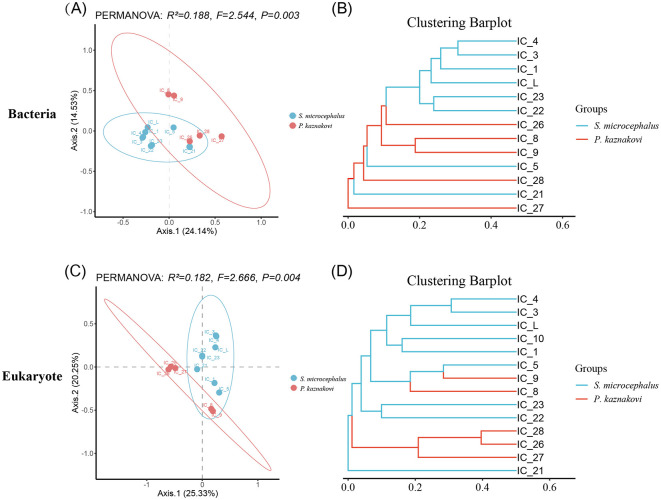
Comparison of beta diversity between *S. microcephalus* and *P. kaznakovi*. Bacteria **(A, B)** and eukaryotes **(C, D)** used PCoA and cluster analysis to assess the distribution differences among the profiles.

In the beta diversity analysis, PCoA analysis (PERMANOVA) based on the Bray-Curtis distance revealed that the gut microbiota and eukaryotic communities were significantly different between two species, with greater similarity observed within species than between species (*R*^2^= 0.188, *P* = 0.003; *R* ^2^= 0.182, *P* = 0.004) (Figures 3A, C). Clustering analysis of both bacteria and eukaryotes demonstrated that all samples from *S. microcephalus* and *P. kaznakovi* were primarily grouped into two distinct branches, exhibiting lower within-group variation and greater between-group sample distances. This finding underscores the differences in bacterial community composition and dietary preferences between *S. microcephalus* and *P. kaznakovi* (Figures 3B, D).

### 3.3 Differences in the composition between *S. microcephalus* and *P. kaznakovi*

**Figure 4:**
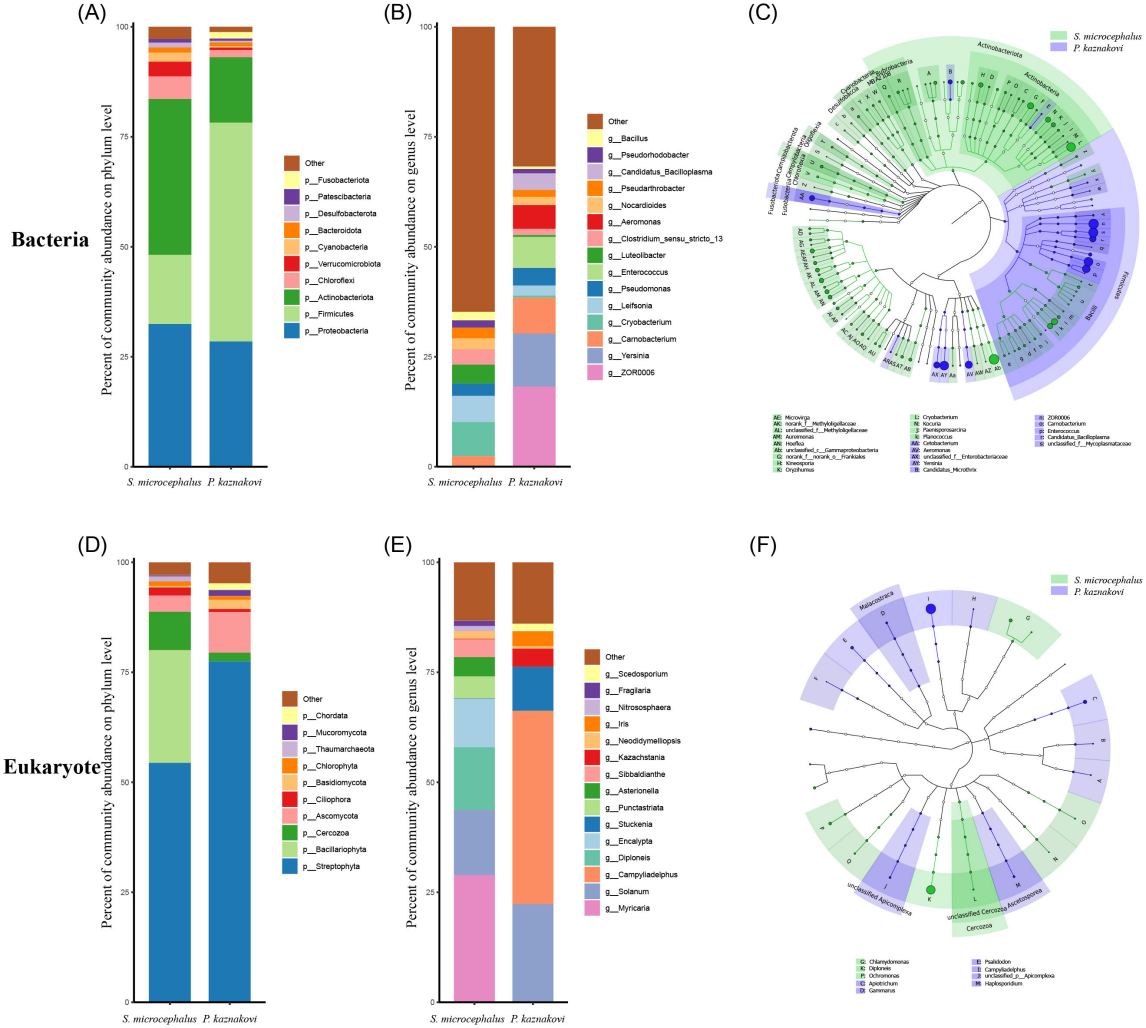
Differences in the composition between *S. microcephalus* and *P. kaznakovi*. **(A, B)** Differences in composition of intestinal bacteria between *S. microcephalus* and *P. kaznakovi* at phylum and genus levels. **(C)** The significant differences in bacterial abundance between *S. microcephalus* and *P. kaznakovi*. **(D, E)** Differences in composition of eukaryotes between *S. microcephalus* and *P. kaznakovi* at phylum and genus levels. **(F)** The significant differences in eukaryotic abundance between *S. microcephalus* and *P. kaznakovi*. The percentage accumulative histogram displays only the taxonomically annotated species. The cladograms generated by LEfSe by non-parametric factorial Kruskal-Wallis rank sum tests and Wilcoxon rank sum tests. Significance at the phylum, class, and genus levels were indicated, with different letters representing significance at the genus level.

In the collected bacterial samples, the top five dominant bacterial phyla detected both in *S. microcephalus* and *P. kaznakovi* were Proteobacteria, Firmicutes, Actinobacteriota, Chloroflexi, and Verrucomicrobiota (Figure 4A and Supplementary Table S1). While the dominant bacterial phyla were consistent across different samples, their relative abundances varied. Analysis of the samples from *S. microcephalus* and *P. kaznakovi* revealed that, excluding unclassified species annotations, both species primarily fed on plant debris (Figure 4D and Supplementary Table S1). Although they shared certain phyla in their diet, such as Streptophyta and Cercozoa, the two species displayed distinct feeding preferences for plants, algae, or zooplankton.

At the phylum level, the bacterial microbiomes of *S. microcephalus* were predominantly composed of Actinobacteriota (35.42%), Proteobacteria (32.44%), and Firmicutes (15.72%) (Figure 4A and Supplementary Table S1). The diet of *S. microcephalus* primarily consisted of Streptophyta (54.41%) and Bacillariophyta (25.65%), with some contributions from Cercozoa (8.67%), Ascomycota (3.70%), and Ciliophora (1.83%) (Figure 4D and Supplementary Table S1). Further analysis at the genus level indicated that *Cryobacterium* (7.72%) and *Leifsonia* (5.98%) were the dominant gut microbes in *S. microcephalus* (Figure 4B and Supplementary Table S1). The dietary composition of *S. microcephalus* included various plants such as *Myricaria* (28.92%), *Solanum* (14.71%), *Encalypta* (11.03%), and *Sibbaldianthe* (4.07%), along with algae including *Diploneis* (14.33%), *Punctastriata* (5.01%), *Asterionella* (4.39%), and *Fragilaria* (1.20%) (Figure 4E and Supplementary Table S1). In contrast, the bacterial microbiomes of *P. kaznakovi* were primarily dominated by Firmicutes (49.72%), followed by Proteobacteria (28.46%) and Actinobacteriota (14.89%) (Figure 4A and Supplementary Table S1). The diet of *P. kaznakovi* was mainly composed of Streptophyta (77.50%), with proportions of Ascomycota (9.25%), Basidiomycota (2.10%), Cercozoa (1.94%), and Chordata (1.53%) (Figure 4D and Supplementary Table S1). At the genus level, *ZOR0006* (18.21%), *Yersinia* (12.05%), *Carnobacterium* (8.17%), *Enterococcus* (7.05%), and *Aeromonas* (5.37%) exhibited high abundance in *P. kaznakovi* (Figure 4B and Supplementary Table S1). The dietary intake of *P. kaznakovi* included plants such as *Campyliadelphus* (43.94%), *Solanum* (22.16%), *Stuckenia* (9.99%), and *Iris* (3.37%), with minimal contributions from algae (Figure 4E and Supplementary Table S1).

Among the top 10 bacterial phyla, the abundance of Actinobacteriota was significantly higher in *S. microcephalus* compared to *P. kaznakovi* (*P* = 0.019), whereas the abundance of Fusobacteriota (*P* = 0.009) and Firmicutes (*P* = 0.006) was significantly higher in *P. kaznakovi* than in *S. microcephalus* (Supplementary Figure 3A). Among the top 15 bacterial genera, *Cryobacterium* (*P* = 0.006) and *Bacillus* (*P* = 0.030) were significantly more abundant in *S. microcephalus* than in *P. kaznakovi*, while *ZOR0006* (*P* = 0.001), *Enterococcus* (*P* = 0.020), *Carnobacterium* (*P* = 0.045), *Candidatus_Bacilloplasma* (*P* = 0.012), and *Aeromonas* (*P* = 0.003) were more abundant in *P. kaznakovi* than in *S. microcephalus* (Supplementary Figure 3B). Additionally, the dietary abundance of *Diploneis* (*P* = 0.026) was significantly higher in *S. microcephalus* compared to *P. kaznakovi*. Conversely, the abundance of *Campyliadelphus* (*P* = 0.045) was significantly higher in *P. kaznakovi* compared to *S. microcephalus* (Supplementary Figure 3C). We further identified specialized bacterial and eukaryotic communities across diverse taxa and generated cladograms from the phylum to genus level using the LEfSe tool. This analysis revealed the community composition from another perspective and identified potential biomarkers. The cladogram analysis indicated that, at the phylum level, Campilobacterota and Actinobacteriota were identified as bacterial biomarkers for *S. microcephalus*, while at the genus level, biomarkers included *Cryobacterium, Planococcus, Paenisporosarcina*, and *Kocuria*, among others (Figure 4C and Supplementary Table S2). In contrast, Fusobacteriota and Firmicutes were identified as bacterial biomarkers at the phylum level for *P. kaznakovi*, while with genus-level biomarkers including *Cetobacterium, Yersinia, ZOR0006, Carnobacterium, Enterococcus*, and others. Additionally, the dietary abundance of Cercozoa was identified as a phylum-level biomarker for *S. microcephalus*, while *Diploneis* and others were recognized as biomarkers at the genus level (Figure 4F and Supplementary Table S2). Furthermore, the dietary abundance of *Gammarus* and other genera were identified as biomarkers for *P. kaznakovi* at the genus level.

### 3.4 Functional metagenomic analysis of intestinal microbiota genes in *S. microcephalus* and *P. kaznakovi*

**Figure 5:**
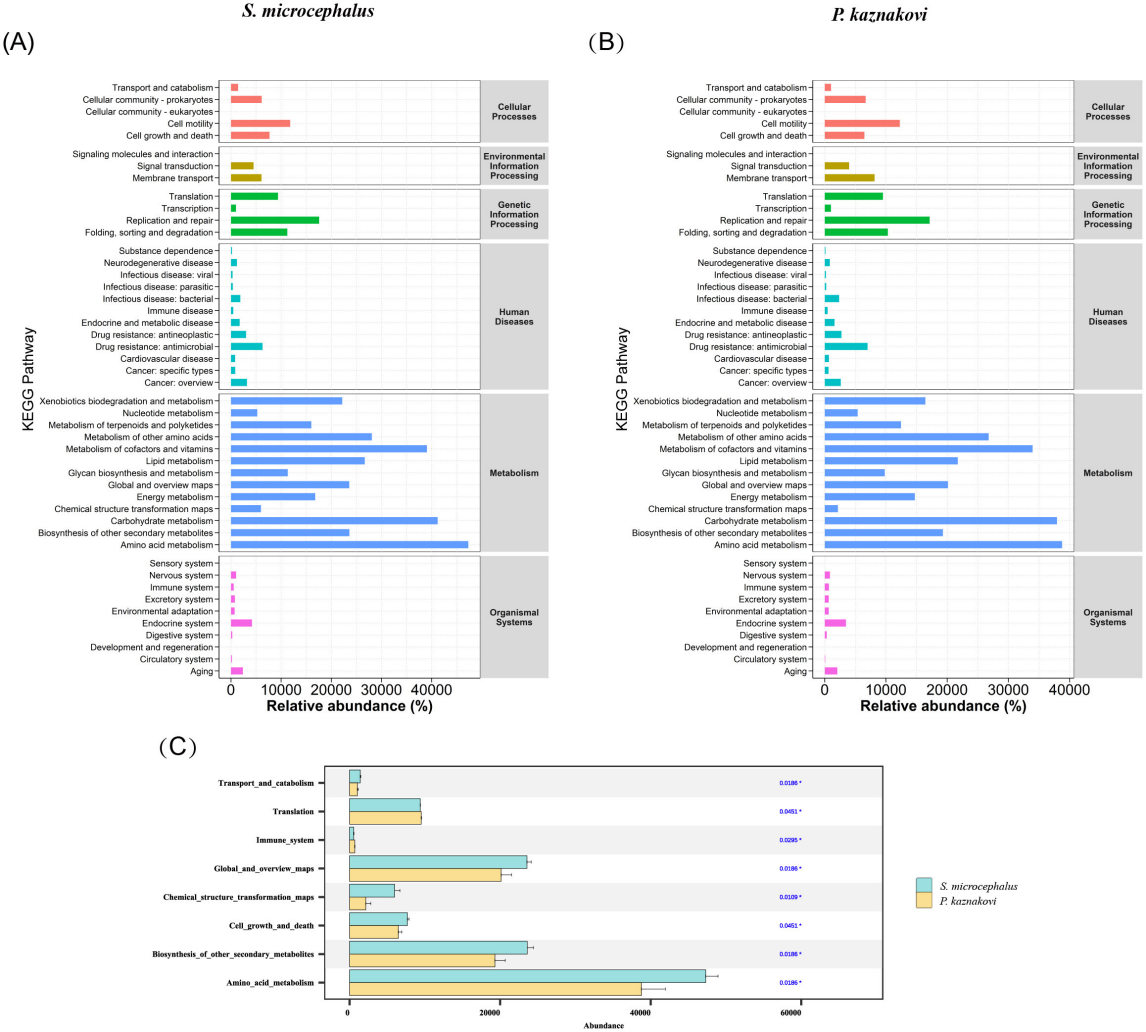
Differences in KEGG pathways between *S. microcephalus* and *P. kaznakovi*. Functional abundance distribution of *S. microcephalus* **(A)** and *P. kaznakovi* **(B)**; **(C)** Results of significance differences in functional abundance between *S. microcephalus* and *P. kaznakovi* based on the Wilcoxon rank-sum test.

The KEGG analysis results for *S. microcephalus* and *P. kaznakovi* revealed differences in the abundance of various microbiota gene functions within the gut, as indicated by pathway 1 and pathway 2 annotation results (Figures 5A, B). Among these pathways, the abundances of Transport and catabolism (*P* = 0.019), Cell growth and death (*P* = 0.045), Global and overview maps (*P* = 0.019), Chemical structure transformation maps (*P* = 0.011), Biosynthesis of other secondary metabolites (*P* = 0.019), and Amino acid metabolism (*P* = 0.019) were significantly higher in *S. microcephalus* compared to *P. kaznakovi* (Figure 5C). In contrast, Translation (*P* = 0.045) and the Immune system (*P* = 0.030) were significantly more abundant in *P. kaznakovi* than in *S. microcephalus*. However, the abundance of metabolic functions in pathway 1 was high for both *S. microcephalus and P. kaznakovi*, followed by genetic information processing and cellular processes. In pathway 2, the three most abundant functions were Amino acid metabolism, Carbohydrate metabolism, and Metabolism of cofactors and vitamins, which are components of the metabolism in pathway 1.

### 3.5 Correlation analysis between gut bacteria and diet in *S. microcephalus* and *P. kaznakovi*

**Figure 6:**
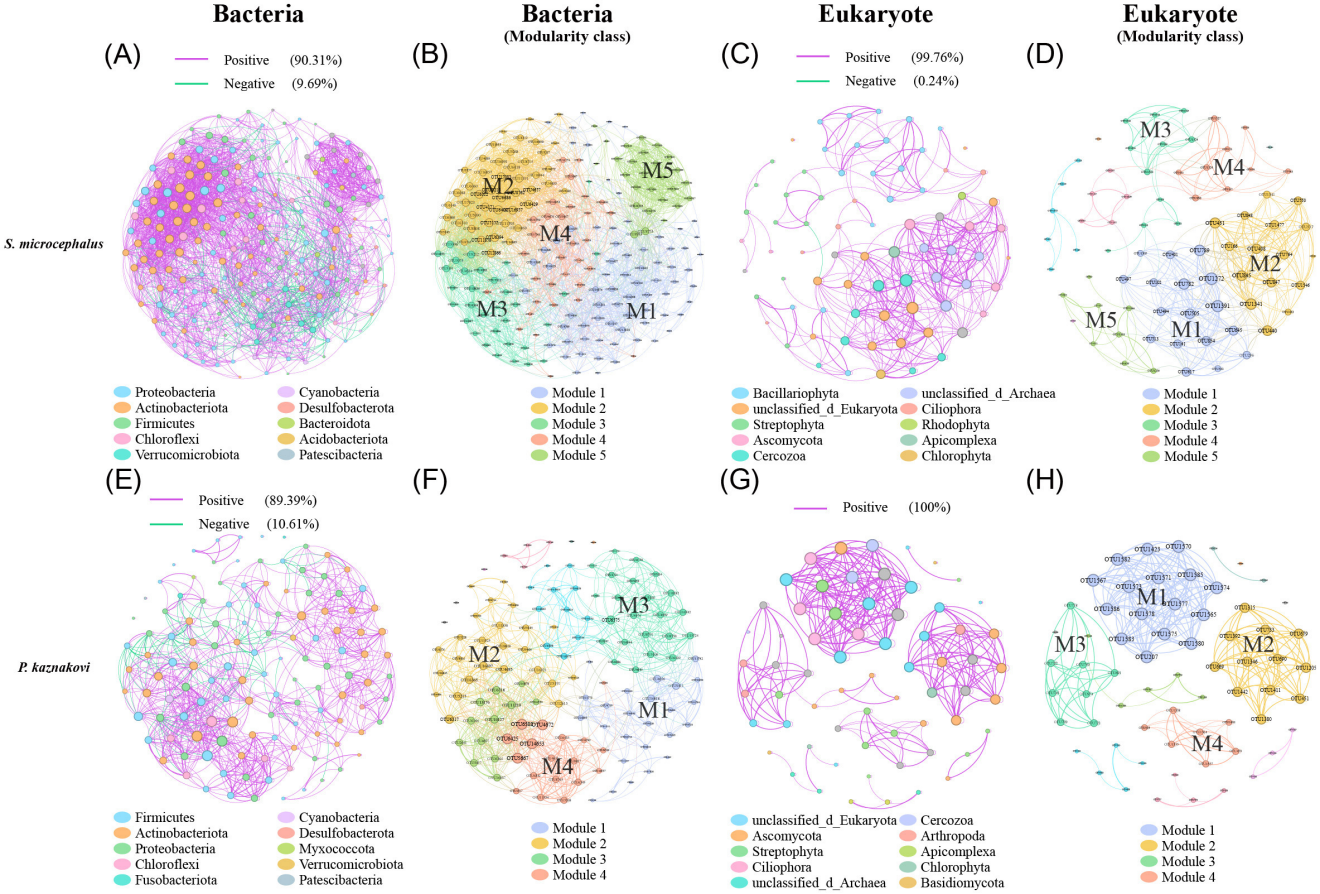
Correlation analysis between gut bacteria and diet in *S. microcephalus* and *P. kaznakovi* based on Spearman's correlation analysis between OTUs. Co-occurrence networks of bacteria in *S. microcephalus* **(A)** and *P. kaznakovi* **(E)**; **(B, F)** are respective modularity class. Co-occurrence networks of eukaryotes in *S. microcephalus* **(C)** and *P. kaznakovi* **(G)**; **(D, H)** are respective modularity class. Each network displays with the top 60 abundances, a correlation coefficient >|0.7|, and a *P* value < 0.05. The nodes were colored by taxonomy at phyla levels. The size of each node is proportional to the number of connections. Each modularity class marks the modules that have a proportion greater than 10%, with M1 having the highest proportion.

The co-occurrence network analysis of the top 60 abundant taxa based on Spearman correlations revealed that *S. microcephalus* exhibited more complex networks than *P. kaznakovi*, both in bacteria (211 nodes and 2818 edges vs. 123 nodes and 914 edges) and eukaryotes (75 nodes and 423 edges vs. 65 nodes and 317 edges) (Supplementary Table S3). Furthermore, nearly all links in the networks were positively correlated across all groups, with a higher proportion of positive correlations observed in eukaryotes compared to bacteria (Figure 6). In the bacterial networks, Proteobacteria, Actinobacteriota, and Firmicutes were the dominant phyla in both *S. microcephalus* and *P. kaznakovi*, constituting 81.04% and 90.25% of the total, respectively (Figures 6A, E and Supplementary Table S4). *S. microcephalus* comprised a total of five modules, while *P. kaznakovi* included 12 modules. However, *S. microcephalus* had five main modules and *P. kaznakovi* had four main modules (defined as modules with a proportion greater than 10%), which accounted for 87.21% and 73.99% of the total, respectively (Figures 6B, F and Supplementary Table S4). In the eukaryotic networks, Bacillariophyta, unclassified_d_Eukaryota, Streptophyta, Ascomycota, and Cercozoa were the dominant phyla in *S. microcephalus*, together representing 74.66%. It had a total of 10 modules, with 5 main modules accounting for 81.34% of the total (Figures 6C, D and Supplementary Table S4). In contrast, *P. kaznakovi* featured unclassified_d_Eukaryota, Ascomycota, Streptophyta, and Ciliophora as the dominant phyla, which together accounted for 59.99%. It had a total of 12 modules, with four main modules accounting for 66.16% of the total (Figures 6G, H and Supplementary Table S4).

### 3.6 Correlation analysis of the gut bacteria and diet combination in *S. microcephalus* and *P. kaznakovi*

**Figure 7:**
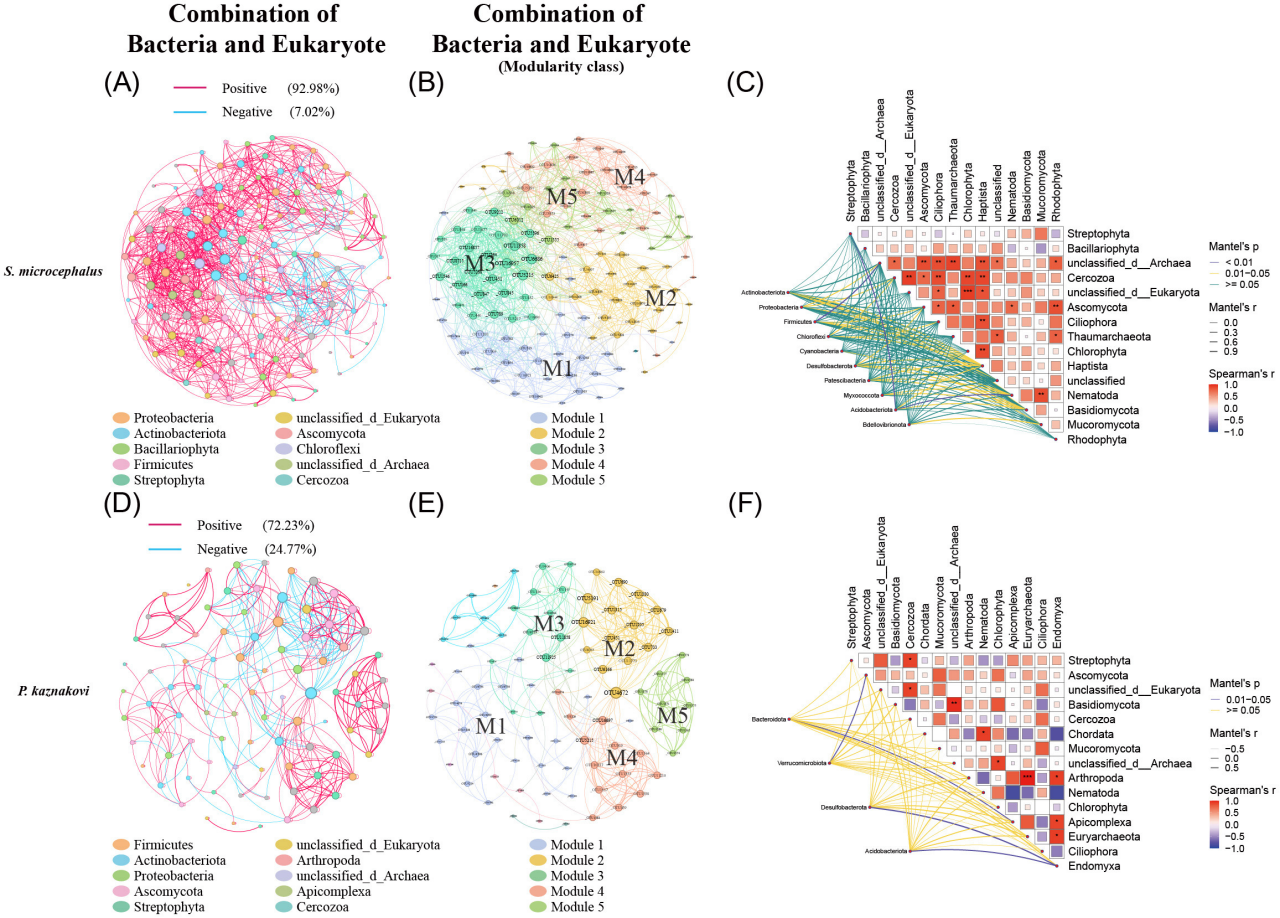
Correlation analysis of the gut bacteria and diet combination in *S. microcephalus* and *P. kaznakovi*. Co-occurrence analysis of the gut microbes and diet in *S. microcephalus* and *P. kaznakovi* based on Spearman's correlation analysis between OTUs. Co-occurrence networks of bacteria and eukaryotes in *S. microcephalus* **(A)** and *P. kaznakovi* **(D)** for the combined analysis; **(B, E)** are respective modularity class. Each network displays with the top 60 abundances, a correlation coefficient >|0.7|, and a *P* value < 0.05. The nodes were colored by taxonomy at phyla levels. The size of each node is proportional to the number of connections. Each modularity class marks the modules that have a proportion greater than 10%, with M1 having the highest proportion. The Mantel test analysis of *S. microcephalus* **(C)** and *P. kaznakovi* **(F)** are based on Spearman's analysis and demonstrates the top 15 abundant phyla with differences.

In the combined analysis of bacteria and eukaryotes, *P. kaznakovi* exhibited the highest proportion of negative correlations (Figure 7D), indicating a greater level of antagonism between gut microbiota and dietary components in this species. In *S. microcephalus*, the dominant taxa included Proteobacteria, Actinobacteriota, Bacillariophyta, and Firmicutes, which collectively accounted for 63.15% of the total, distributed across six modules, with five main modules comprising 99.24% of the total (Figures 7A, B and Supplementary Table S4). Conversely, in *P. kaznakovi*, the dominant taxa included Firmicutes, Actinobacteriota, Proteobacteria, Ascomycota, and Streptophyta, amounting to 65.16%, with a total of 10 modules and five main modules representing 83.15% of the total (Figures 7D, E and Supplementary Table S4). Furthermore, based on Spearman analysis and considering the top 15 abundant phyla, supplemented by the Mantel test revealed that more significant correlations between gut bacteria and eukaryotes in *S. microcephalus* (Figures 7C, F and Supplementary Table S5). For example, Actinobacteriota displayed significant correlations with Haptista (*P* = 0.038), Nematoda (*P* = 0.033), and Basidiomycota (*P* = 0.019). Additionally, the diet of *S. microcephalus*, which includes Haptista, Nematoda, and Basidiomycota, showed the highest number of significant correlations with its gut bacterial phyla. In *P. kaznakovi*, significant correlations were observed between Bacteroidota and Apicomplex (*P* = 0.033), Verrucomicrobiota and Ascomycota (*P* = 0.042), as well as between Desulfobacterota and Acidobacteriota with Endomyxa (*P* = 0.017 for both).

According to the modular networks, modules with a degree of 20 or more, as well as interactions between bacteria and eukaryotes in *S. microcephalus* are primarily represented by M1, M3, and M5 (Figure 7B and Supplementary Table S6). In M1, the abundance of *Planococcus* in the phylum Firmicutes exhibited a positive correlation with *Chlamydomonas* of Chlorophyta and *Amphora* of Bacillariophyta. In M3, the abundance of *Nocardioides* in the phylum Actinobacteriota showed a positive correlation with *Chaetomium* of Ascomycota and *Diacronema* of Haptista. The abundance of *Skermanella* in Proteobacteria and *Nocardioides* in Actinobacteriota both showed a positive correlation with *Arthrinium, Neodidymelliopsis*, and *Cordyceps* of Ascomycota. Additionally, the abundance of *Blastococcus* in Actinobacteriota showed a positive correlation with *Neodidymelliopsis* of Ascomycota. The abundance of *Nocardioides* in Actinobacteriota showed a positive correlation with *Pyropia* of Rhodophyta. In M5, the abundance of *Hyphomicrobium* in Proteobacteria showed a positive correlation with *Rhynchospora* of Streptophyta. Modules with a degree of 10 or more and interactions between bacteria and eukaryotes in *P. kaznakovi* are mainly M2, M3, and M4 (Figure 7E and Supplementary Table S6). In M2, the abundance of *Cryobacterium* in Actinobacteriota, *Planomicrobium* in Firmicutes, and *Hyphomicrobium* in Proteobacteria exhibited a negative correlation with *Scedosporium, Mycocalicium*, and *Thelebolus* of Ascomycota. In M3, the abundance of *Enterococcus* and *Romboutsia* in Firmicutes showed a negative correlation with *Solanum* of Streptophyta and *Kazachstania* of Ascomycota; however, *Nocardioides* in Actinobacteriota and *Ralstonia* in Proteobacteria exhibited a positive correlation with these two foods. In M4, the abundance of *Solibacillus* in Firmicutes, *Nocardioides* in Actinobacteriota, and *ZOR0006* in Firmicutes showed a positive correlation with *Stuckenia, Achillea, Rhynchospora* of Streptophyta, and *Psalidodon* of Chordata.

## 4 Discussion

The diversity and abundance of fish microbiota are influenced by a variety of factors. Eternal factors primarily include the water environment, while internal factors encompass the host and feeding habits (Yi et al., 2019; Pan et al., 2023; Degregori et al., 2024). For fish species that share overlapping distribution areas, the feeding habits of the host serve as the major influencing factor. A study has indicated that the diversity of intestinal microflora is higher in omnivorous fish, following the order: omnivorous > herbivorous > plankton feeder > carnivorous (Jiao et al., 2023; Yu et al., 2024). This is attributed to the gut of phytophagous fish, which is rich in cellulose-degrading microbes, resulting in a higher abundance of intestinal microbes. In our study, *S. microcephalus* exhibited higher dietary diversity compared to *P. kaznakovi*, and the α-diversity of the gut microbiome was also higher in *S. microcephalus* than in *P. kaznakovi*. Furthermore, β-diversity analyses revealed that different species cluster separately, suggesting that the gut microbial composition within the same species is consistent and may be closely associated with its host and dietary habits (Brooks et al., 2016; Aizpurua et al., 2021).

In terms of gut microbes, whole-metagenome shotgun sequencing analysis revealed that Proteobacteria, Firmicutes, Actinobacteria, and Bacteroidetes are the predominant phyla in yellowfin sea bream (Pan et al., 2021). A 16S rDNA analysis of the endemic fish species Schizothorax o'connori in Tibet identified Proteobacteria, Verrucomicrobia, Firmicutes, Bacteroidetes, and Actinobacteriota as the dominant phyla (Shang et al., 2019). Firmicutes are present in similar proportions across herbivorous, carnivorous, omnivorous, and filter-feeding species (Liu et al., 2016). In our study, we found that Proteobacteria, Firmicutes, and Actinobacteriota are the dominant bacteria in *S. microcephalus* and *P. kaznakovi*. Furthermore, both fish species share the same top five most abundant bacterial phyla, which may indicate a process of convergent evolution, reflecting similar ecological adaptations, and evolutionary strategies in response to their habitats. Additionally, wild animals typically exhibit adaptability, such as the ability to safely consume rotting, pathogen-infected meat, and toxic plants (Huang et al., 2016; Lanszki et al., 2018), which accounts for the presence of numerous harmful bacteria in their gut. *Aeromonas*, recognized as a common harmful microorganism in fish, increases in abundance when zebrafish are infected with *Aeromonas hydrophila* (Yang et al., 2017). In our study, we observed a significantly higher abundance of *Aeromonas* in *P. kaznakovi* compared to *S. microcephalus*. This finding may be closely associated with the specific dietary sources consumed by *P. kaznakovi*.

Due to the scarcity of biological resources on the plateau, various fish species have gradually evolved distinct mouth shapes, positions, and feeding habits to adapt to their environments and available food sources, thereby achieving niche differentiation. Prolonged evolutionary processes have resulted in differences in mouth position among Schizothorax fish, including inferior and subinferior placement, which are closely associated with their feeding habits. Previous studies indicate that *S. microcephalus* possesses either an inferior or subinferior mouth position, while *P. kaznakovi* has an inferior position (Wu and Wu, 1992). Although the mouth positions of these two species are similar, the blunt and rounded snout of *P. kaznakovi* enhances its ability to capture smaller aquatic animals. We found that both species primarily compete for Streptophyta, Cercozoa, and Ciliophora at the phylum level; however, variations in their feeding proportions reflect differences in resource utilization. *S. microcephalus* also consumes Bacillariophyta and small amounts of Cercozoa and Ciliophora, whereas *P. kaznakovi* includes small amounts of Cercozoa and Chordata in its diet, suggesting a more carnivorous tendency in *P. kaznakovi* compared to *S. microcephalus*. A study employing Stable Isotope Analysis has demonstrated significant competition for dietary resources and niche differentiation among sympatric fish species across different water layers (Pelage et al., 2022). The division of feeding resources between these two species may be attributed to competitive interactions and adaptive strategies within the same watershed (Cicala et al., 2024). However, in contrast to previous studies, these two fish species did not heavily consume aquatic insects, a phenomenon that may be explained by the scarcity of food resources or seasonal changes (Bereded et al., 2021). During the summer, fish migration for spawning and other factors lead to increased energy expenditure, prompting fish to select food that is more readily available and abundant. Consequently, the diets of both fish species primarily consist of plant-based foods during this season.

In terms of gut microbes and diet, it has been reported that the abundance of Firmicutes and Actinobacteriota exhibits a negative correlation with altitude (Bereded et al., 2022). However, in our study, both *S. microcephalus* and *P. kaznakovi* were found to inhabit the same altitude, yet Firmicutes were significantly more abundance in *P. kaznakovi* compared to *S. microcephalus*, while Actinobacteriota displayed the opposite trend. Moreover, previous studies have indicated that *Cetobacterium* is the most abundant species in carnivorous channel catfish and largemouth bass (Larsen et al., 2014). Notably, compared to fishmeal (FM), *Cetobacterium* was found to be more abundant in fish fed diets containing black soldier fly (BSF), likely due to its close association with the digestion processes in carnivorous fish (Foysal and Gupta, 2022). Interestingly, our study identified *Cetobacterium* as a biomarker for the gut microbiota of *P. kaznakovi*. Its presence may enhance the utilization of meat resources by *P. kaznakovi*, reduce competition for resources, and improve overall resource utilization efficiency. Additionally, the dietary analysis of *P. kaznakovi* revealed that its diet contained a substantial amount of *Campyliadelphus*, a plant that exhibited a higher abundance of Firmicutes in autumn (Ma et al., 2017). This suggests that fish may ingest microorganisms these parasitize in plants during feeding, leading to an increased abundance of Firmicutes. Therefore, the abundance of Firmicutes at the phylum level and *Cetobacterium* at the genus level is easily influenced by diet (Suhr et al., 2023). These findings further illustrate that *P. kaznakovi* is an omnivorous fish with carnivorous tendencies, while *S. microcephalus* is an omnivorous fish with herbivorous inclinations.

In addition, our findings indicate that the gut microbiota demonstrates the highest expression levels related to metabolism, attributed to their capacity to metabolize proteins, and complex carbohydrates while producing a vast array of metabolites (Yoo et al., 2020). Furthermore, they play a vital role in immune regulation within the intestinal mucosa (Wang et al., 2024). Notably, the gut microbiota of *P. kaznakovi* exhibits higher levels of immune system expression. This phenomenon may represent a mechanism that has evolved in the host over time, enabling adaptation to environmental or dietary changes through the modulation of gut microbial communities, and the associated immune responses.

For most species, feeding habits are closely linked to the composition of gut microbiota (Chen et al., 2020). In our study, we observed a predominantly positive relationship between gut microbiota and diet, with *S. microcephalus* exhibiting more positive correlations than *P. kaznakovi*. Negative correlations were relatively rare, likely due to the mutualistic symbiosis between most gut microbes and their hosts, which reflects a converging feeding ecology that enhances adaptation to the host's intestinal environment (Parmentier et al., 2024). In *P. kaznakovi*, the abundance of *Solibacillus* and *ZOR0006* within the Firmicutes is positively correlated with animal-derived food, specifically Chordata. This correlation may arise from their presence facilitating the digestion of proteins and fats (Cho and Lee, 2020). In contrast, *Planococcus* in *S. microcephalus* is more effective for the digestion of algae, such as *Chlamydomonas* and *Amphora*. Actinobacteria play a crucial role in breaking down plant and fungal cell walls (Chater, 2016). We observed that *Nocardioides* of Actinobacteria was primarily positively correlated with Ascomycota in *S. microcephalus*, whereas in *P. kaznakovi, Nocardioides* was mainly positively correlated with Streptophyta. These findings suggest that *Nocardioides* may play a key role in the degradation and utilization of various food sources, including *Arthrinium, Neodidymelliopsis, Cordyceps, Stuckenia, Achillea*, and *Rhynchospora*. Additionally, both fish species exhibited multiple dietary categories that are not significantly correlated with Proteobacteria, potentially due to their diverse physiological functions, ability to utilize a wide range of carbon sources, and significant role in energy accumulation within the host (Lu et al., 2012). The richness of carbon sources in plants could also contribute to the minimal impact of dietary categories on the composition of Proteobacteria. However, the relationship between gut microbiota and dietary intake is complex and influenced by numerous factors. Despite considerable advances in our understanding, many aspects of the interaction between diet and gut microbiota remain to be elucidated.

## 5 Conclusions

This study characterized the composition, diversity, functionality, and interactions with feeding habits of the gut microbiota in two fish species, *S. microcephalus* and *P. kaznakovi*. Both fish species possessed distinct core gut microbiota but shared several dominant bacterial phyla, including Firmicutes, Proteobacteria, and Actinobacteriota. Despite inhabiting the same aquatic environment and being classified as omnivorous, the two species exhibited different feeding preferences. Specifically, *S. microcephalus* primarily consumed Streptophyta and Bacillariophyta at the phylum level and *Myricaria, Solanum*, and *Diploneis* at the genus level. In contrast, *P. kaznakovi* predominantly fed on Streptophyta at the phylum level and *Campyliadelphus, Solanum*, and *Stuckenia* at the genus level. Additionally, a complex relationship was observed between gut microbiota and feeding habits, with multiple microbial taxa showing significant correlations with diet. This study provides valuable insights into the relationship between gut microbiota and feeding habits, contributing to a better understanding of the ecological niche differentiation between theses fish species.

## Data Availability

The raw reads for this study can be found in the NCBI BioProject (Accession Number: PRJNA1139086), accessible at https://www.ncbi.nlm.nih.gov/bioproject.
