# Supplementary material for: Differences and correlation analysis of feeding habits and intestinal microbiome in Schizopygopsis microcephalus and Ptychobarbus kaznakovi in the upper reaches of Yangtze River
Source: Front Microbiol. 2025 Mar 11;16:1513401. doi: 10.3389/fmicb.2025.1513401 (PMC11935114; doi:10.3389/fmicb.2025.1513401)
Supplement: Supplementary file 1 [file Data_Sheet_1.docx]

**Differences and correlation analysis of feeding habits and intestinal microbiome in *Schizopygopsis microcephalus* and *Ptychobarbus kaznakovi* in the upper reaches of Yangtze River**


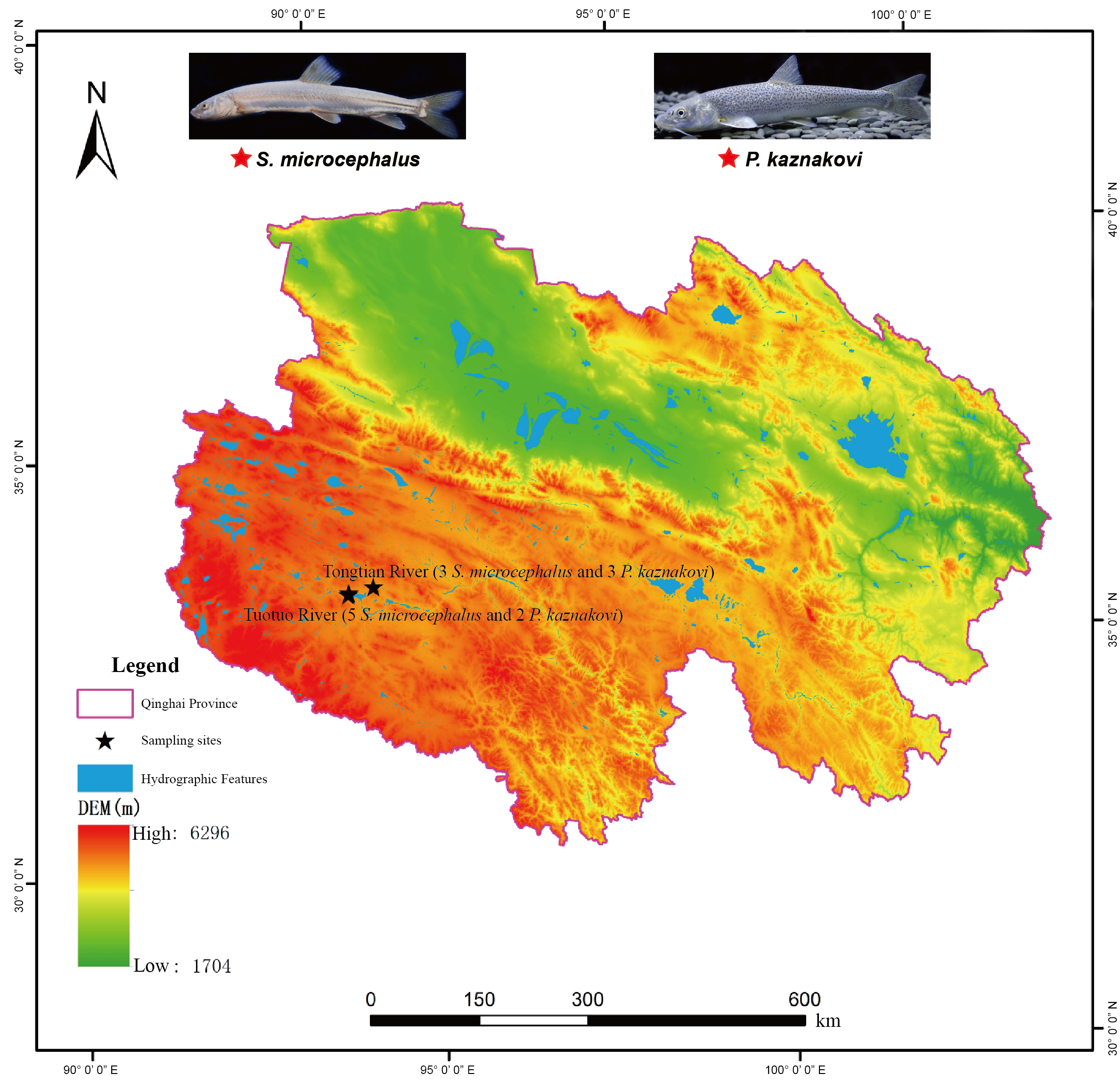


**Supplementary Figure 1** The geographic locations of sampling sites for fish species *S. microcephalus* and *P. kaznakovi* in Qinghai Province, China, including the Tuotuo River and the Tongtian River.


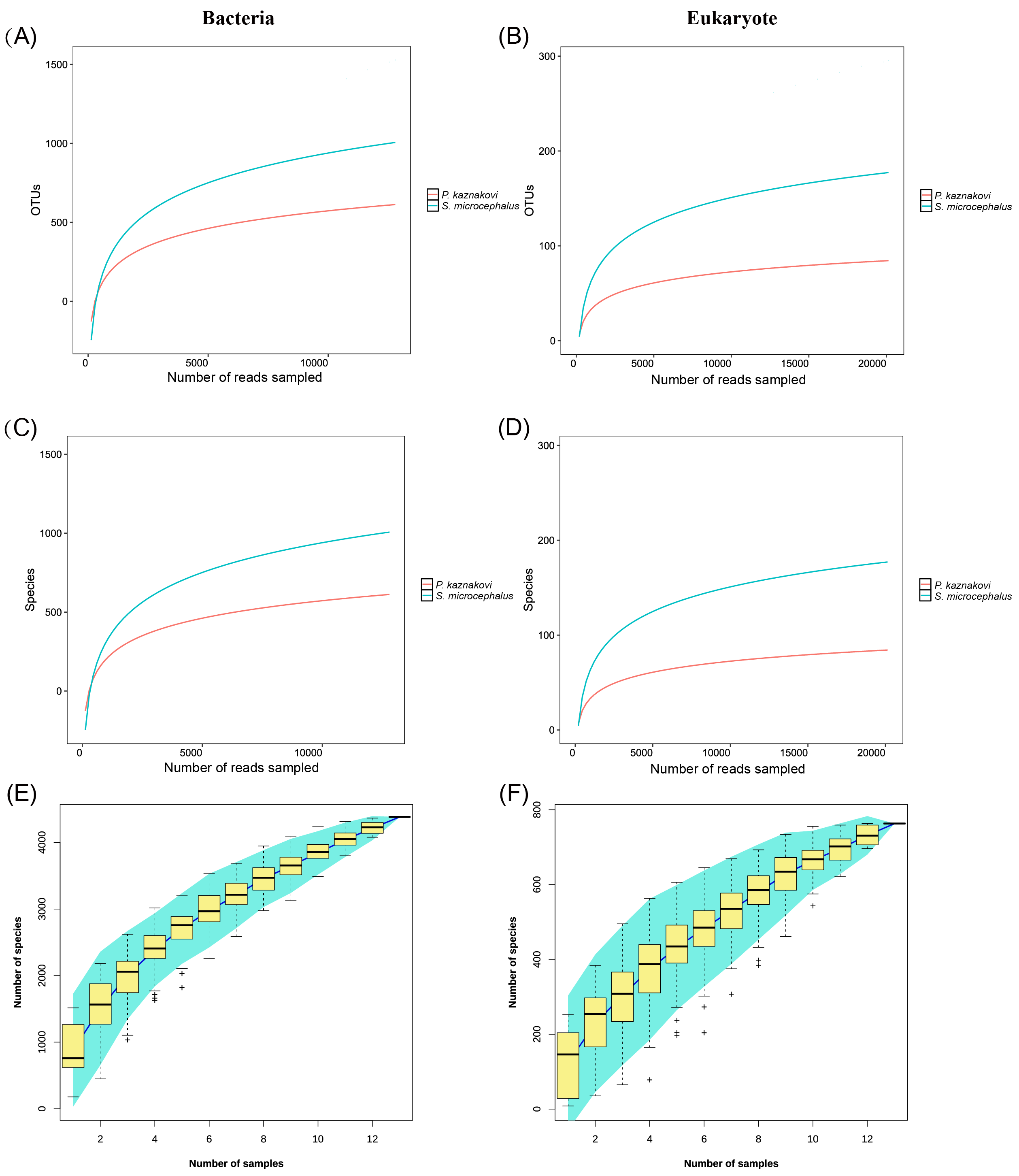


**Supplementary Figure 2** The analysis of sequencing depth and sample size for gut microbiota and eukaryotes in *S. microcephalus* and *P. kaznakovi*. Rarefaction curves of gut microbiota based on OTUs **(A)** and Species **(C)**. Rarefaction curves of eukaryotes based on OTUs **(B)** and Species **(D)**. **(E)** Accumulation of species based on the number of microbiota samples. **(F)** Accumulation of species based on the number of eukaryotes samples.


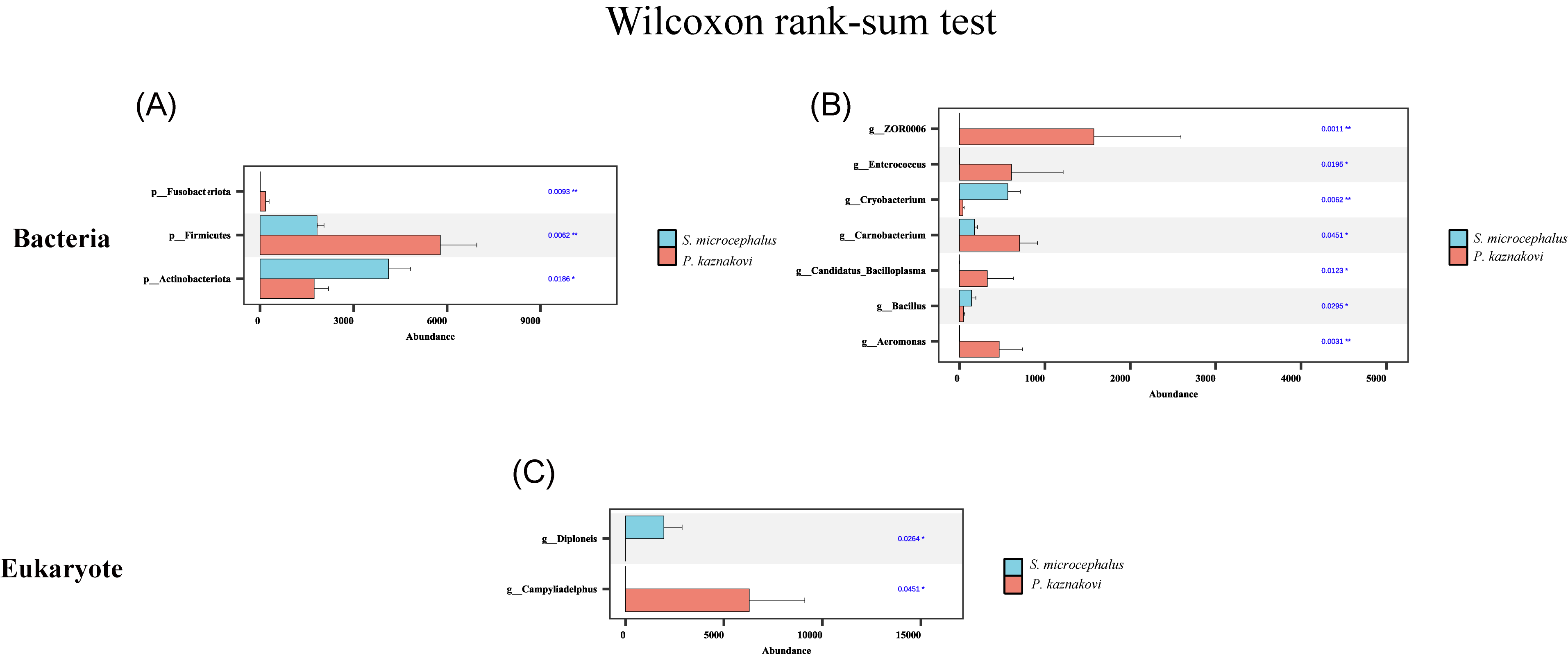


**Supplementary Figure 3** The significant differences in the phylum and genus levels of gut microbiota bacteria and eukaryotes between *S. microcephalus* and *P. kaznakovi*. Significant differences in the top ten phyla **(A)** and top fifteen genera **(B)** of bacteria. Significant differences in the top fifteen genera **(C)** of eukaryotes.

**Supplementary Table 1** Differences in the composition of phyla and genera between *S. microcephalus* and *P. kaznakovi*. The top 10 phyla and top 15 genera of intestinal bacteria and eukaryotes are presented, respectively. The following data were obtained after removing unclassified species annotations.

|  | **16S rRNA (Bacteria)** | | **18S rRNA (Eukaryote)** | |
| --- | --- | --- | --- | --- |
|  | ***S. microcephalus*** | ***P. kaznakovi*** | ***S. microcephalus*** | ***P. kaznakovi*** |
| **Phylum (Top10)** | Actinobacteriota (35.42%) | Firmicutes (49.72%) | Streptophyta (54.41%) | Streptophyta (77.50%) |
|  | Proteobacteria (32.44%) | Proteobacteria (28.46%) | Bacillariophyta (25.65%) | Ascomycota (9.25%) |
|  | Firmicutes (15.72%) | Actinobacteriota (14.89%) | Cercozoa (8.67%) | Basidiomycota (2.10%) |
|  | Chloroflexi (5.16%) | Chloroflexi (1.62%) | Ascomycota (3.70%) | Cercozoa (1.94%) |
|  | Verrucomicrobiota (3.31%) | Fusobacteriota (1.48%) | Ciliophora (1.83%) | Chordata (1.53%) |
|  | Cyanobacteria (2.07%) | Bacteroidota (0.80%) | Thaumarchaeota (1.14%) | Mucoromycota (1.35%) |
|  | Bacteroidota (1.18%) | Verrucomicrobiota (0.57%) | Chlorophyta (1.03%) | Chlorophyta (0.81%) |
|  | Desulfobacterota (1.10%) | Patescibacteria (0.51%) | Basidiomycota (0.37%) | Ciliophora (0.66%) |
|  | Patescibacteria (0.78%) | Desulfobacterota (0.41%) | Mucoromycota (0.26%) | Thaumarchaeota (0.06%) |
|  | Fusobacteriota (0.02%) | Cyanobacteria (0.34%) | Chordata (0.07%) | Bacillariophyta (0.03%) |
|  | Other (2.80%) | Other (1.20%) | Other (2.87%) | Other (4.77%) |
| **Genus** **(Top15)** | Cryobacterium (7.72%) | ZOR0006 (18.21%) | Myricaria (28.92%) | Campyliadelphus (43.94%) |
|  | Leifsonia (5.98%) | Yersinia (12.05%) | Solanum (14.71%) | Solanum (22.16%) |
|  | Luteolibacter (4.30%) | Carnobacterium (8.17%) | Diploneis (14.33%) | Stuckenia (9.99%) |
|  | Clostridium_sensu_stricto_13 (3.46%) | Enterococcus (7.05%) | Encalypta (11.03%) | Kazachstania (4.15%） |
|  | Pseudomonas (2.78%） | Aeromonas (5.37%) | Punctastriata (5.01%) | Iris (3.37%) |
|  | Nocardioides (2.53%) | Pseudomonas (4.00%) | Asterionella (4.39%) | Scedosporium (1.70%) |
|  | Pseudarthrobacter (2.40%) | Candidatus_Bacilloplasma (3.77%) | Sibbaldianthe (4.07%) | Neodidymelliopsis (0.55%) |
|  | Carnobacterium (2.39%) | Leifsonia (2.28%) | Neodidymelliopsis (1.69%) | Myricaria (0.14%) |
|  | Bacillus (1.93%) | Nocardioides (1.87%) | Nitrososphaera (1.20%) | Nitrososphaera (0.06%) |
|  | Pseudorhodobacter (1.66%) | Pseudarthrobacter (1.60%) | Fragilaria (1.20%) | Diploneis (0.00%) |
|  | Yersinia (0.01%) | Clostridium_sensu_stricto_13 (1.43%) | Kazachstania (0.11%) | Encalypta (0.00%) |
|  | Aeromonas (0.01%) | Pseudorhodobacter (0.99%) | Stuckenia (0.07%) | Punctastriata (0.00%) |
|  | Enterococcus (0.01%) | Bacillus (0.55%) | Scedosporium (0.07%) | Asterionella (0.00%) |
|  | Candidatus_Bacilloplasma (0.01%) | Cryobacterium (0.46%) | Campyliadelphus (0.00%) | Sibbaldianthe (0.00%) |
|  | ZOR0006 (0.01%) | Luteolibacter (0.41%) | Iris (0.00%) | Fragilaria (0.00%) |
|  | Other (64.81%） | Other (31.78%） | Other (13.23%） | Other (13.94%） |

**Supplementary Table 2** Cladograms representing the biomarkers of intestinal microbiota bacteria and eukaryotes at the genus level for *S. microcephalus* and *P. kaznakovi*.

|  | **ID** | **Genus** | **ID** | **Genus** | **ID** | **Genus** |
| --- | --- | --- | --- | --- | --- | --- |
| **Bacteria** | AA | Cetobacterium | A | norank_f__norank_o__Actinomarinales | a | Desulfobacca |
|  | AB | Skermanella | B | Candidatus_Microthrix | b | Desulfocapsa |
|  | AC | norank_f__Amb-16S-1323 | C | norank_f__norank_o__0319-7L14 | c | Syntrophotalea |
|  | AD | Methylobacterium-Methylorubrum | D | norank_f__Euzebyaceae | d | Anaerobacillus |
|  | AE | Microvirga | E | Longivirga | e | Bacillus |
|  | AF | Salinarimonas | F | norank_f__Sporichthyaceae | f | Halobacillus |
|  | AG | Norank_f__Beijerinckiaceae | G | norank_f__norank_o__Frankiales | g | Oceanobacillus |
|  | AH | unclassified_f__Beijerinckiaceae | H | Kineosporia | h | Paraliobacillus |
|  | AI | unclassified_f__Devosiaceae | I | Georgenia | i | Jeotgalibacillus |
|  | AJ | Filomicrobium | J | Demequina | j | Paenisporosarcina |
|  | AK | norank_f__Methyloligellaceae | K | Oryzihumus | k | Planococcus |
|  | AL | unclassified_f__Methyloligellaceae | L | Cryobacterium | l | unclassified_f__Planococcaceae |
|  | AM | Aureimonas | M | unclassified_f__Microbacteriaceae | m | Salipaludibacillus |
|  | AN | Hoeflea | N | Kocuria | n | ZOR0006 |
|  | AO | Bauldia | O | Nitriliruptor | o | Carnobacterium |
|  | AP | Pseudolabrys | P | norank_f__Nitriliruptoraceae | p | Enterococcus |
|  | AQ | norank_f__norank_o__Rhizobiales | Q | norank_f__norank_o__norank_c__MB-A2-108 | q | unclassified_o__Lactobacillales |
|  | AR | Defluviimonas | R | Rubrobacter | r | Candidatus_Bacilloplasma |
|  | AS | Roseovarius | S | Gillisia | s | unclassified_f__Mycoplasmataceae |
|  | AT | Rubellimicrobium | T | norank_f__norank_o__0319-6G20 | t | Cohnella |
|  | AU | Yoonia-Loktanella | U | Sulfurovum | u | Paenibacillus |
|  | AV | Aeromonas | V | norank_f__JG30-KF-CM45 | v | norank_f__norank_o__RsaHf231 |
|  | AW | Thiocapsa | W | Synechocystis_PCC-6803 | w | norank_f__Eubacteriaceae |
|  | AX | unclassified_f__Enterobacteriaceae | X | Oscillatoria_PCC-6304 | x | Faecalibacterium |
|  | AY | Yersinia | Y | Planktothrix_NIVA-CYA_15 | y | Lutispora |
|  | AZ | Halomonas | Z | unclassified_o__Babeliales | z | Pelosinus |
|  | Aa | Psychrobacter |  |  |  |  |
|  | Ab | unclassified_c__Gammaproteobacteria |  |  |  |  |
| **Eukaryote** | A | unclassified_f__Debaryomycetaceae | G | Chlamydomonas | M | Haplosporidium |
|  | B | Pichia | H | Prototheca | N | Telaepolella |
|  | C | Apiotrichum | I | Campyliadelphus | O | unclassified_o__Euglyphida |
|  | D | Gammarus | J | unclassified_p__Apicomplexa | P | Ochromonas |
|  | E | Psalidodon | K | Diploneis | Q | Woloszynskia |
|  | F | Myxobolus | L | unclassified Cercozoa |  |  |

**Supplementary Table 3** Data on the correlation analysis of network co-occurrence for *S. microcephalus* and *P. kaznakovi*. The nodes, edges, average degree, and modularity in the table are based on data derived from the top 60 abundant taxa.

|  | **Bacteria** | | **Eukaryote** | | **Combination of Both** | |
| --- | --- | --- | --- | --- | --- | --- |
|  | ***S. microcephalus*** | ***P. kaznakovi*** | ***S. microcephalus*** | ***P. kaznakovi*** | ***S. microcephalus*** | ***P. kaznakovi*** |
| **Nodes** | 211 | 123 | 75 | 65 | 133 | 89 |
| **Edges** | 2818 | 914 | 423 | 317 | 1140 | 432 |
| **Avg. Degree** | 26.711 | 14.862 | 11.28 | 9.754 | 17.143 | 9.708 |
| **Modularity** | 0.516 | 0.574 | 0.558 | 0.663 | 0.534 | 0.723 |
| **Graph Density** | 0.127 | 0.122 | 0.152 | 0.152 | 0.13 | 0.11 |
| **Avg. Clustering Coefficient** | 0.618 | 0.696 | 0.778 | 0.939 | 0.655 | 0.786 |
| **Avg. Path Length** | 2.574 | 3.224 | 3.338 | 1.068 | 2.784 | 3.516 |

**Supplementary Table 4** Information on the nodes and modules proportions of bacterial, eukaryotic, and bacterial-eukaryotic co-occurrence networks in *S. microcephalus* and *P. kaznakovi*. Co-occurrence analysis based on Spearman’s correlation analysis between OTUs. Nodes represent species taxonomic annotations at the top ten phyla.

|  | **Bacteria** | | **Eukaryote** | | **Combination of Both** | |
| --- | --- | --- | --- | --- | --- | --- |
|  | ***S. microcephalus*** | ***P. kaznakovi*** | ***S. microcephalus*** | ***P. kaznakovi*** | ***S. microcephalus*** | ***P. kaznakovi*** |
| **Nodes** | Proteobacteria (33.65%) | Firmicutes (31.71%) | Bacillariophyta (24.00%) | unclassified_d_Eukaryota (20.00%) | Proteobacteria (21.80%) | Firmicutes (16.85%) |
|  | Actinobacteriota (30.33%) | Actinobacteriota (30.08%) | unclassified_d_Eukaryota (17.33%) | Ascomycota (18.46%) | Actinobacteriota (20.30%) | Actinobacteriota (13.48%) |
|  | Firmicutes (17.06%) | Proteobacteria (28.46%) | Streptophyta (14.67%) | Streptophyta (15.38%) | Bacillariophyta (11.28%) | Proteobacteria (13.48%) |
|  | Chloroflexi (4.74%) | Chloroflexi (4.07%) | Ascomycota (9.33%) | Ciliophora (6.15%) | Firmicutes (9.77%) | Ascomycota (11.24%) |
|  | Verrucomicrobiota (3.32%) | Fusobacteriota (1.63%) | Cercozoa (9.33%) | unclassified_d_Archaea (4.62%) | Streptophyta (6.02%) | Streptophyta (10.11%) |
|  | Cyanobacteria (2.84%) | Cyanobacteria (0.81%) | unclassified_d_Archaea (8.00%) | Cercozoa (4.62%) | unclassified_d_Eukaryota (5.26%) | unclassified_d_Eukaryota (7.87%) |
|  | Desulfobacterota (1.42%) | Desulfobacterota (0.81%) | Ciliophora (8.00%) | Arthropoda (4.62%) | Ascomycota (3.76%) | Arthropoda (3.37%) |
|  | Bacteroidota (0.95%) | Myxococcota (0.81%) | Rhodophyta (1.33%) | Apicomplexa (3.08%) | Chloroflexi (3.76%) | unclassified_d_Archaea (2.55%) |
|  | Acidobacteriota (0.95%) | Verrucomicrobiota (0.81%) | Apicomplexa (1.33%) | Chlorophyta (3.08%) | unclassified_d_Archaea (3.01%) | Apicomplexa (2.55%) |
|  | Patescibacteria (0.95%) | Patescibacteria (0.81%) | Chlorophyta (1.33%) | Basidiomycota (3.08%) | Cercozoa (3.01%) | Cercozoa (2.55%) |
| **Modules** | M1 (34.60%) | M1 (20.33%) | M1 (22.67%) | M1 (24.62%) | M1 (25.56%) | M1 (26.97%) |
|  | M2 (22.27%) | M2 (20.33%) | M2 (21.33%) | M2 (18.46%) | M2 (21.05%) | M2 (16.85%) |
|  | M3 (15.17%) | M3 (18.70%) | M3 (14.67%) | M3 (12.31%) | M3 (20.30%) | M3 (14.61%) |
|  | M4 (15.17%) | M4 (14.63%) | M4 (12.00%) | M4 (10.77%) | M4 (18.80%) | M4 (14.61%) |
|  | M5 (12.80%) | M5 (9.76%) | M5 (10.67%) | M5 (7.69%) | M5 (13.53%) | M5 (10.11%) |
|  |  | M6 (8.94%) | M6 (8.00%) | M6 (7.69%) | M6 (0.75%) | M6 (6.74%) |
|  |  | M7 (3.25%) | M7 (6.67%) | M7 (6.15%) |  | M7 (4.49%) |
|  |  | M8 (0.81%) | M8 (1.33%) | M8 (4.62%) |  | M8 (2.25%) |
|  |  | M9 (0.81%) | M9 (1.33%) | M9 (3.08%) |  | M9 (2.25%) |
|  |  | M10 (0.81%) | M10 (1.33%) | M10 (1.54%) |  | M10 (1.12%) |
|  |  | M11 (0.81%) |  | M11 (1.54%) |  |  |
|  |  | M12 (0.81%) |  | M12 (1.54%) |  |  |

**Supplementary Table 5** Mantel test analysis of *S. microcephalus* and *P. kaznakovi* based on Spearman's analysis, displaying significant correlation information within the top 15 abundant phyla with differences.

|  | **Spec** | **Env** | **Mantel's r** | **Mantel's p** |
| --- | --- | --- | --- | --- |
| ***S. microcephalus*** | Actinobacteriota | Basidiomycota | 0.831 | 0.019 |
|  | Actinobacteriota | Nematoda | 0.924 | 0.033 |
|  | Actinobacteriota | Haptista | 0.945 | 0.038 |
|  | Proteobacteria | Cercozoa | 0.491 | 0.019 |
|  | Proteobacteria | unclassified_d__Archaea | 0.639 | 0.042 |
|  | Proteobacteria | Haptista | 0.770 | 0.044 |
|  | Firmicutes | unclassified_d__Archaea | 0.855 | 0.007 |
|  | Firmicutes | Haptista | 0.767 | 0.031 |
|  | Firmicutes | Ascomycota | 0.565 | 0.032 |
|  | Firmicutes | unclassified | 0.753 | 0.041 |
|  | Chloroflexi | Haptista | 0.846 | 0.027 |
|  | Chloroflexi | Ciliophora | 0.595 | 0.033 |
|  | Chloroflexi | Cercozoa | 0.459 | 0.045 |
|  | Cyanobacteria | Mucoromycota | 0.326 | 0.025 |
|  | Desulfobacterota | Ascomycota | 0.635 | 0.022 |
|  | Desulfobacterota | Haptista | 0.918 | 0.026 |
|  | Desulfobacterota | Basidiomycota | 0.783 | 0.030 |
|  | Desulfobacterota | Nematoda | 0.874 | 0.046 |
|  | Patescibacteria | Basidiomycota | 0.811 | 0.013 |
|  | Patescibacteria | Nematoda | 0.866 | 0.021 |
|  | Patescibacteria | Haptista | 0.762 | 0.037 |
|  | Myxococcota | Nematoda | 0.629 | 0.026 |
|  | Myxococcota | Basidiomycota | 0.580 | 0.032 |
|  | Bdellovibrionota | Mucoromycota | 0.776 | 0.044 |
|  | Acidobacteriota | Streptophyta | 0.489 | 0.008 |
|  | Acidobacteriota | Haptista | 0.492 | 0.014 |
|  | Acidobacteriota | Basidiomycota | 0.577 | 0.016 |
|  | Acidobacteriota | Nematoda | 0.595 | 0.005 |
| ***P. kaznakovi*** | Bacteroidota | Apicomplexa | 0.423 | 0.033 |
|  | Verrucomicrobiota | Ascomycota | 0.552 | 0.042 |
|  | Desulfobacterota | Endomyxa | 0.891 | 0.017 |
|  | Acidobacteriota | Endomyxa | 0.668 | 0.017 |

**Supplementary Table 6** The relationship between bacteria and eukaryotes in each module of the modular co-occurrence networks. *S. microcephalus* showed the data with a degree above 20 in each module. *P. kaznakovi* showed the data with a degree above 10 in each module.

| ***S. microcephalus*** | | | | | | | | | |
| --- | --- | --- | --- | --- | --- | --- | --- | --- | --- |
|  |  | **Bacteria** | | | | **Eukaryote** | | | |
| **Modules** | **Correlation** | **OTU ID** | **Phylum** | **Genus** | **Degree** | **OTU ID** | **Phylum** | **Genus** | **Degree** |
| **M1** | Positive | OTU16921 | Firmicutes | Planococcus | 23 | _OTU782  _OTU617  _OTU513  _OTU505  _OTU1391  _OTU1303  _OTU1230 | Cercozoa  Chlorophyta  Cercozoa  unclassified_d__Eukaryota  unclassified_d__Eukaryota  unclassified_d__Eukaryota  Bacillariophyta | unclassified_o__Glissomonadida  Chlamydomonas  unclassified_p__Cercozoa  Ochromonas  unclassified_f__Ochromonadaceae  unclassified_d__Eukaryota  Amphora | 21  22  21  23  26  20  20 |
| **M2** |  | ------ | | | | | | | |
| **M3** | Positive  Positive  Positive  Positive  Positive  Positive  Positive  Positive | OTU8715  OTU6686  OTU5268  OTU5217  OTU5215  OTU16957  OTU16890  OTU16857 | Proteobacteria  Actinobacteriota  Proteobacteria  Proteobacteria  Actinobacteriota  Chloroflexi  Firmicutes  Chloroflexi | norank_f__Methyloligellaceae  norank_f__Ilumatobacteraceae  unclassified_f__Methyloligellaceae  Skermanella  Nocardioides  norank_f__norank_o__norank_c__Gitt-GS-136  unclassified_f__Planococcaceae  norank_f__norank_o__norank_c__KD4-96 | 28  37  29  27  37  37  22  30 | _OTU789 | Apicomplexa | Cryptosporidium | 29 |
|  | Positive  Positive  Positive  Positive  Positive  Positive  Positive | OTU9212  OTU8715  OTU5596  OTU5268  OTU16957  OTU16857  OTU11858 | Actinobacteriota  Proteobacteria  Actinobacteriota  Proteobacteria  Chloroflexi  Chloroflexi  Actinobacteriota | norank_f__norank_o__Microtrichales  norank_f__Methyloligellaceae  norank_f__Euzebyaceae  unclassified_f__Methyloligellaceae  norank_f__norank_o__norank_c__Gitt-GS-136  norank_f__norank_o__norank_c__KD4-96  Nocardioides | 30  28  30  29  37  30  34 | _OTU517 | Ascomycota | Chaetomium | 21 |
|  | Positive  Positive  Positive | OTU9212  OTU5596  OTU11858 | Actinobacteriota  Actinobacteriota  Actinobacteriota | norank_f__norank_o__Microtrichales  norank_f__Euzebyaceae  Nocardioides | 30  30  34 | _OTU440 | Haptista | Diacronema | 25 |
|  | Positive  Positive  Positive  Positive  Positive  Positive  Positive  Positive  Positive | OTU8715  OTU6686  OTU5268  OTU5217  OTU5215  OTU16957  OTU16890  OTU16857  OTU11858 | Proteobacteria  Actinobacteriota  Proteobacteria  Proteobacteria  Actinobacteriota  Chloroflexi  Firmicutes  Chloroflexi  Actinobacteriota | norank_f__Methyloligellaceae  norank_f__Ilumatobacteraceae  unclassified_f__Methyloligellaceae  Skermanella  Nocardioides  norank_f__norank_o__norank_c__Gitt-GS-136  unclassified_f__Planococcaceae  norank_f__norank_o__norank_c__KD4-96  Nocardioides | 28  37  29  27  37  37  22  30  34 | _OTU432 | Ascomycota | Arthrinium | 24 |
|  | Positive  Positive  Positive  Positive  Positive  Positive  Positive  Positive | OTU6686  OTU5268  OTU5217  OTU5215  OTU16957  OTU16857  OTU11858  OTU11793 | Actinobacteriota  Proteobacteria  Proteobacteria  Actinobacteriota  Chloroflexi  Chloroflexi  Actinobacteriota  Actinobacteriota | norank_f__Ilumatobacteraceae  unclassified_f__Methyloligellaceae  Skermanella  Nocardioides  norank_f__norank_o__norank_c__Gitt-GS-136  norank_f__norank_o__norank_c__KD4-96  Nocardioides  Blastococcus | 37  29  27  37  37  30  34  26 | _OTU1546 | Ascomycota | Neodidymelliopsis | 29 |
|  | Positive  Positive  Positive  Positive  Positive  Positive  Positive | OTU8715  OTU6686  OTU5268  OTU5215  OTU16957  OTU16857  OTU11858 | Proteobacteria  Actinobacteriota  Proteobacteria  Actinobacteriota  Chloroflexi  Chloroflexi  Actinobacteriota | norank_f__Methyloligellaceae  norank_f__Ilumatobacteraceae  unclassified_f__Methyloligellaceae  Nocardioides  norank_f__norank_o__norank_c__Gitt-GS-136  norank_f__norank_o__norank_c__KD4-96  Nocardioides | 28  37  29  37  37  30  34 | _OTU1541 | Rhodophyta | Pyropia | 20 |
|  | Positive  Positive  Positive  Positive  Positive  Positive  Positive  Positive  Positive  Positive | OTU9212  OTU8715  OTU6686  OTU5596  OTU5268  OTU5217  OTU5215  OTU16957  OTU16857  OTU11858 | Actinobacteriota  Proteobacteria  Actinobacteriota  Actinobacteriota  Proteobacteria  Proteobacteria  Actinobacteriota  Chloroflexi  Chloroflexi  Actinobacteriota | norank_f__norank_o__Microtrichales  norank_f__Methyloligellaceae  norank_f__Ilumatobacteraceae  norank_f__Euzebyaceae  unclassified_f__Methyloligellaceae  Skermanella  Nocardioides  norank_f__norank_o__norank_c__Gitt-GS-136  norank_f__norank_o__norank_c__KD4-96  Nocardioides | 30  28  37  30  29  27  37  37  30  34 | _OTU1477 | Ascomycota | Cordyceps | 25 |
| **M4** |  | ------ | | | | | | | |
| **M5** | Positive  Positive  Positive | OTU8765  OTU6588  OTU6012 | Proteobacteria  Chloroflexi  Proteobacteria | Hyphomicrobium  norank_f__JG30-KF-CM45  norank_f__norank_o__norank_c__Alphaproteobacteria | 21  25  31 | _OTU1555 | Streptophyta | Rhynchospora | 30 |

| ***P. kaznakovi*** | | | | | | | | | |
| --- | --- | --- | --- | --- | --- | --- | --- | --- | --- |
|  |  | **Bacteria** | | | | **Eukaryote** | | | |
| **Modules** | **Correlation** | **OTU ID** | **Phylum** | **Genus** | **Degree** | **OTU ID** | **Phylum** | **Genus** | **Degree** |
| **M1** |  | ------ | | | | | | | |
| **M2** | Negative  Negative  Negative  Positive  Negative  Negative  Negative | OTU4672  OTU5191  OTU16921  OTU6166  OTU11773  OTU10962  OTU8765 | Actinobacteriota  Actinobacteriota  Firmicutes  Proteobacteria  Firmicutes  Firmicutes  Proteobacteria | Cryobacterium  unclassified_f__Micromonosporaceae  Planococcus  unclassified_f__Enterobacteriaceae  Clostridium_sensu_stricto_13  Planomicrobium  Hyphomicrobium | 23  20  20  16  14  12  12 | _OTU1205  _OTU690  _OTU733  _OTU451  _OTU679  _OTU1315  _OTU1380 | Ascomycota  Endomyxa  Arthropoda  Ascomycota  Ascomycota  unclassified_d__Eukaryota  Ascomycota | Scedosporium  Haplosporidium  Gammarus  Mycocalicium  Thelebolus  unclassified_d__Eukaryota  unclassified_f__Debaryomycetaceae | 16  16  16  16  16  16  16  16 |
| **M3** | Negative  Positive  Positive  Negative | OTU11925  OTU11858  OTU9406  OTU8723 | Firmicutes  Actinobacteriota  Proteobacteria  Firmicutes | Enterococcus  Nocardioides  Ralstonia  Romboutsia | 15  15  12  11 | _OTU126  _OTU145 | Streptophyta  Ascomycota | Solanum  Kazachstania | 12  12 |
| **M4** | Positive  Positive | OTU16697  OTU5215 | Firmicutes  Actinobacteriota | Solibacillus  Nocardioides | 16  16 | _OTU1558  _OTU1557  _OTU459  _OTU1564  _OTU1555 | Streptophyta  Chordata  Nematoda  Streptophyta  Streptophyta | Stuckenia  Psalidodon  unclassified_p__Nematoda  Achillea  Rhynchospora | 13  13  13  13  13 |
|  | Positive  Positive | OTU11210  OTU10127 | Firmicutes  Firmicutes | ZOR0006  unclassified_c__Bacilli | 13  13 | _OTU1558  _OTU1557  _OTU459  _OTU1564  _OTU1555  _OTU1481 | Streptophyta  Chordata  Nematoda  Streptophyta  Streptophyta  Ascomycota | Stuckenia  Psalidodon  unclassified_p__Nematoda  Achillea  Rhynchospora  Penicillium | 13  13  13  13  13  11 |
| **M5** |  | ------ | | | | | | | |
